# In-depth analysis of the medical supply for indigenous people in North-Eastern Colombia: a dominance of infectious diseases and only insufficient therapeutic options

**DOI:** 10.1186/s13690-024-01338-w

**Published:** 2024-07-31

**Authors:** Hannah Bauer, Hagen Frickmann, Gustavo Concha, Lothar Kreienbrock, Maria Hartmann, Philipp Warnke, Ralf Matthias Hagen, Ernst Molitor, Achim Hoerauf, Simone Kann

**Affiliations:** 1 Julius-Maximilians University, Würzburg, 97070 Germany; 2 Department of Microbiology and Hospital Hygiene, Bundeswehr Hospital Hamburg, Hamburg, 20359 Germany; 3 Institute for Medical Microbiology, Virology and Hygiene, University Medicine Rostock, Rostock, 18057 Germany; 4 Organization Wiwa Yugumaiun Bunkanarrua Tayrona (OWYBT), Valledupar, 200001 Colombia; 5 Department of Biometry, Epidemiology and Information Processing, University of Veterinary Medicine Hannover, Hannover, 30559 Germany; 6 Department of Microbiology and Hospital Hygiene, Bundeswehr Central Hospital Koblenz, Koblenz, 56070 Germany; 7 Institute of Medical Microbiology, Immunology and Parasitology (IMMIP), University Hospital Bonn, Bonn, 53127 Germany

**Keywords:** Epidemiology, Health services, Indigenous, Therapeutics, Medical neglect

## Abstract

**Background:**

Colombian indigenous Wiwas are exposed to a variety of partly complex medical conditions with a predominance of infectious diseases. The study provided here aims at verifying of falsifying previous suspicions on therapeutic shortcomings and neglect of disease categories.

**Material and methods:**

Local diagnoses within various subpopulations of indigenous Wiwas obtained by a study physician and local health brigades and health points between 2017 and 2018 were coded following the ICD 10 classification from 2019. Proportions of diagnoses per ICD-10 sub-chapter were evaluated to find diseases and to rank the occurrence of diagnoses in the population of indigenous people. Thereafter, the available medication provided by the indigenous health care provider Dusakawi for the treatment of the indigenous patients was analyzed in regard of its sufficiency to cover the recorded diseases.

**Results:**

The majority of the diseases found in the communities cannot at all (32%) or only partially (56%) be treated according to available guidelines. Only few (12%), predominantly infectious diseases, were covered completely by the provided medication. Notably, there are some ICD chapters with diseases that do only rarely appear at all in the gained datasets, e.g., complications during birth, mental disorders or cancer.

**Conclusions:**

An expansion and revision of the medical supply for the indigenous population of the Sierra Nevada de Santa Marta is needed. An emergency kit for medical brigades and health points should be provided and in place. Awareness for neglected diseases needs to be created.

**Supplementary Information:**

The online version contains supplementary material available at 10.1186/s13690-024-01338-w.

**Table:** 

Text Box 1. Contributions to the literature
• There is limited evidence on the quality of medical care for Colombian indigenous individuals
• Present health care offers for this population neglect relevant diagnosis groups, resulting in shortcomings of the provided medical care and in associated health risks.
• A broadening of diagnostic and therapeutic offers should be considered to overcome those observed shortcomings.

## Introduction

Resource-poor indigenous individuals are highly vulnerable to severe medical consequences of basically treatable or even curable diseases [1]. As previously suggested [2], Colombian indigenous populations don’t make an exemption from this rule. Consequently, our research team felt a need to analyze potential therapeutic shortcomings as well as neglected diseases in Colombian indigenous Wiwas as suspected in a previous assessment [3]. Although the focus of previous studies on the Colombian Wiwa population was on gastrointestinal infections and Chagas disease [3–15], many other diseases affecting the local indigenous with a persisting predominance of infectious diseases were found and recorded as well [2]. Within such a previous analysis [2], the question arose whether or not the provided medication by a locally active health care provider Dusakawi would be efficient or not to cover all the diseases found. To better judge this, a composite dataset was aimed to establish comprising subsets from on-site assessments as well as from health points and health brigades, working in the zones of the indigenous habitats. Based on such a dataset, obtained diagnoses were meant to be compared with a medication list representing the only accessible medication for the Wiwas.

As repeatedly reported by our group [2–15], the indigenous tribe called Wiwa lives in isolated areas in the Sierra Nevada de Santa Marta, north-east of Colombia. Although a few villages have a small health point in their area, they are only sparsely equipped with medical devices and medication and are not permanently medically staffed. Therefore, if Wiwa patients suffer from any medical complaint, they usually have to walk to the next health point, implying an up to six hours walking distance. If in-patient treatment is needed, the necessary way to reach it can be even longer.

Medical care for the Wiwas is ensured by the adoption of Law 691 in Colombia, regulating basic medical care for indigenous people in 2001 [16]. Within the Colombian health system, the needs of indigenous people are addressed by the Sistema General de Seguridad en Social e Salud (SGSS). Therein, the indigenous people belong to the so-called subsidiary regime, which supports the poorest parts of the population and is financed through general taxes. This entitles the patients for basic care only [16], which includes, e.g. an X-ray assessment, but, e.g. no pacemaker operation. As the health care provider Dusakawi is both aware of medical needs as well as of general necessities including lacking personal and diagnostic capacities, they try to improve this situation by sending health brigades to the villages sporadically, for a 1–2 day(s) stay each. The medical brigades consist of a physician, an auxiliary nurse, an odontologist and an auxiliary dental nurse. Their focus is on preventive medicine, e.g., vaccinations, but they also take care of reported medical complaints for the time of their stay. This is of course a help for the people, however, for a detailed analysis of the complaints, including diagnostic options for differential diagnoses, this is not sufficient. Even follow ups, longer assistance, and further tests, etc. are out of reach [2]. Therefore, the therapy is symptom-based and often fails to cure the underlying diseases. This results in the risk of missing severe underlying medical conditions, of developing complications and sequelae or even in death. In addition, the medication that can be provided is limited and so, therapeutic options for complex diseases are often not in place. Even more, diseases like hypertension or others that need a long-term observation to find the correct dosage and combination of drugs, cannot be adequately taken care of.

In the previous study quoted above, we gained information about common diseases in the area of interest with a strong dominance on infectious diseases [2]. Further, an initial superficial analysis had given hints for insufficient therapeutic options as well as neglect of several disease categories [2]. To further verify or falsify those initial impressions, we provide an in-depth analysis focusing subjects based on additional data sources. In line with this, the aim of this study is the analysis whether and to what extent the available medication is sufficient to treat the recorded diseases and whether there is evidence for a neglect of disease categories. To the best of our knowledge, comparable studies specifically addressing the quality of medical care for Colombian indigenous people on a holistic level have not been provided so far.

## Materials and methods

### Ethical approval

The field study was approved by the Ethics Committee of Santa Marta, Colombia (Acta No 032018). The written informed consent was obtained from each participant or from the parent or legal guardian of a child prior to participation. The study was performed in accordance with the principles of the Declaration of Helsinki.

The data, provided by Dusakawi, could not be assigned to any patient anymore, as it was provided from Dusakawi in an anonymized way. The indigenous Ethic committee board of the Organization Wiwa Yugumaiun Bunkanarrua Tayrona, which is the institution leading Dusakawi, approved and authorized the use of the data. The handling of the data was performed in accordance with the principles of the Declaration of Helsinki.

### Study design

The study was conducted as an observational cross-sectional study comparing medical diagnoses of Colombian indigenous people called Wiwa and medication available for their medical treatment. By doing so, potential mismatches between accessible medical care and actual medical needs were assessed. The information on the medical diagnoses were partly provided by a local medical care provided called Dusakawi and – as a quality control procedure – partly set by a study physician.

### Data collection and medical information

Three diagnostic data sets were included in the analysis. The first data set was collected by a study physician during a program against Chagas Disease in 2017 and 2018 [2], that we performed ourselves. In the following, this is named ST for “study data”. Summarized, examinations of indigenous patents were offered in the four villages Tezhumake, Cherua, (Department Cesar), Ashintukwa, and Seminke, (Department La Guajira) during the study period.

The second and third data sets were provided by Dusakawi and gathered by its health brigades (DB = Dusakawi Health Brigades) and the Dusakawi health points in Valledupar, Becerril, Codazzi, La Paz and San Juan (HP = Dusakawi Health Points). From those datasets, the years 2017 and 2018 were likewise assessed [2] coming from the same villages that had been included into the study. The health point in Valledupar is located on a hospital campus and so that severe cases can directly be referred to the hospital. The other health points are located in the Sierra. Accordingly, the patients have to walk to the next hospital for many hours.

All data was coded according to the International Statistical Classification of Diseases and Related Health Conditions System (ICD 10) from 2019. Chapter XXI (Factors influencing health status and contact with health services) was not included in the assessment, because this chapter does not incorporate diagnoses. For tuberculosis and HIV, special programs are available. For this reason, respective data were not excluded as well.

The medication available is provided by Dusakawi to the physicians in charge of caring for the villages and health points, e.g., when they visit the villages as health brigades. Each physician takes the mediation box with him- or herself. After use, it is refilled in one of Dusakawi´s outlet points. For the composition of the medical kit, please see Table 1. The health points have only small amounts of prescription free drugs available, if at all. If a nurse is available, he or she gives this medication to the patients in need. If a nurse is not in place, the health point is closed and medication cannot be received.

**Table 1:** Summarized list of the medications available for the therapy of the Wiwas provided by Dusakawi, ordered in alphabetic categories (More details can be freely accessed via the supplementary materials of the previously published work [2])

Category	Medication
Anaesthetic	Lidocaine chlorohydrate
Analgesic	Paracetamol, Diclofenac, Metamizole, Ibuprofen, Naproxen + Caffeine, Naproxen, Tramadolst
Antianginosum	Isosorbide, Isosorbide dinitrate
Antiarrhythmic drug	Amiodarone, Beta-methyldigoxin
Anti-asthmathic drug	Aminophylline, Ipratropium bromide, Salbutamol, Theophylline
Antibiotic	Nalidixic acid, Amikacin, Amoxicillin, Ampicillin, Azithromycin, Cefalexin, Cefradine, Ceftriaxone, Ciprofloxacin, Clarithromycin, Colistin + Neomycin Dicloxacillin, Doxycycline, Erythromycin, Gentamicin, Metronidazole, Neomycin + Polymyxin + Dexamethasone, Nitrofurantoin, Nitrofurazone Norfloxacin, Oxacillin, Penicillin, Cotrimoxazole, Sucralfate
Anticoagulant	Warfarin sodica
Antidementive	Nimodipine
Antidepressant	Amitriptyline, Fluoxetine, Trazodone
Antidiabetic drug	Glibenclamide, Metformin
Antidot	Anatoxina tetanica
Antiemetic	Metoclopramide
Antiepileptic/Anticonvulsant	Valproate, Carbamazepine, Phenytoin
Antifibrinolytic	Tranexamic acid
Antihelminthic	Albendazole, Mebendazole, Pyrantel pamoate
Antihistaminic	Chlorphenamine, Cromoglicate, Diphenhydramine, Dimenhydrinate, Hydroxyzine, Ketotifen, Loratadine, Ranitidine
Antihypertensive	Alpha methyldopa, Amlodipine, Captopril, Carvedilol, Clonidine chlorhydrate, Clopidogrel, Enalapril, Losartan, Losartan + , Hydrochlorothiazide, Metoprolol, Nifedipine, Propranolol, Timolol maleate Valsartan, Valsartan + Hydrochlorothiazide, Verapamil
Antimalarial/ DMARD	Chloroquine
Antimycotic	Clotrimazole, Fluconazole, Ketoconazole, Nystatin + Zinc oxide, Nystatin
Antiparkinsonian	Amantadine (Virustatic), Biperiden, Levodopa + Carbidopa
Antiplatelet drug	Acetylsalicylic acid
Antiprotozoal	Tinidazole
Antipsychotic	Haloperidol, Levomepromazine, Olanzapine, Pipotiazine, Risperidone
Antiscabiosic	Benzyl benzoate, Crotamiton
Antitussive	Dihydrocodeine
Carminative	Aluminium hydroxide + Magnesium + Simeticone
Desinfection	Silver sulphadiazine
Diuretic	Spironolactone, Furosemide, Hydrochlorothiazide
Fluids	Sodium chloride, Hartmann solution, Physiological serum
Glucocorticoid	Beclomethasone, Betamethasone, Dexamethasone, Hydrocortisone, Hydrocortisone + Benzocaine, Methylprednisolone, Prednisolone, Betamethasone + Clotrimazole + Neomycin
Gout medicine	Colchicine
Hormone	Conjugated Oestrogens, Levonorgestrel, Levonorgestrel + Ethinylestradiol, Levothyroxine, Medroxyprogesterone + Oestradiol, Medroxyprogesterone acetate
Immunosuppressant/DMARD	Methotrexate
Lipid-lowering drug	Atorvastatin, Gemfibrozil
Migraine medication	Ergotamine + Caffeine
Muscle relaxant	Vecuronium bromide, Methocarbamol
Parasympatholytic	Atropine (sulphate)
Prokinetic	Triembutine
Proton pump inhibitors	Esomeprazole, Omeprazole
Spasmolytic	Butylscopalamine bromide (Hioszinbutyl bromide)
Sympathomimetic	Adrenaline, Brimonidine (tartrate), Oxymetazoline
Uricostatic	Allopurinol
Virustatics	Acyclovir
Vitamin/Trace mineral/supplement	Ascorbic acid, Folic acid, Calcium + Vitamin D, Potassium chloride, Iron fumarate + folic acid + ascorbic acid (vitamin C), Salts for oral rehydration, Zinc sulphate, Iron sulphate, Vitamin B1 (Thiamine), Vitamin A (Retinol), Vitamin B12, Vitamin K

### Analysis of the medication list

The collected diagnoses from the ST dataset and data from the DB/HP datasets were aligned and compared with the medication available. Therefore, the following order of the assessments was applied: First, recommendations of national/international guidelines were considered. If those were not available, information about treatments was collected from expert societies and textbook-chapters. A comprehensive overview of the references consulted to decide on therapeutical correctness is provided as a supplementary material 1. Based on those references, the decision on the appropriateness of available medication was performed by medical experts.

Treatable diagnoses were classified as such if both, first-line and second-line therapy were available (marked in green in the Tables 2 and 3). If this was not the case and only basic drugs without alternatives were available, (e.g. no replacement for penicillin in case of a penicillin allergy), or if some therapy components could not be provided (e.g., *Entamoeba histolytica* trophozoite therapy with metronidazole/tinidazole is in place, but the decontamination of cysts with paromomycin is not feasible) or therapies could not address common complications (e.g., bleedings), diagnoses were classified as partially treatable (marked in yellow in the Tables 2 and 3). If important first-line therapy was missing at all, a diagnosis was classified as not treatable (e.g., no heparin available for acute myocardial infarction, no insulin for patients with diabetes I, no urapidil or nitro-glycerine for the treatment of a hypertensive crisis, etc.) (marked in red in the Tables 2 and 4).

**Table 2:**
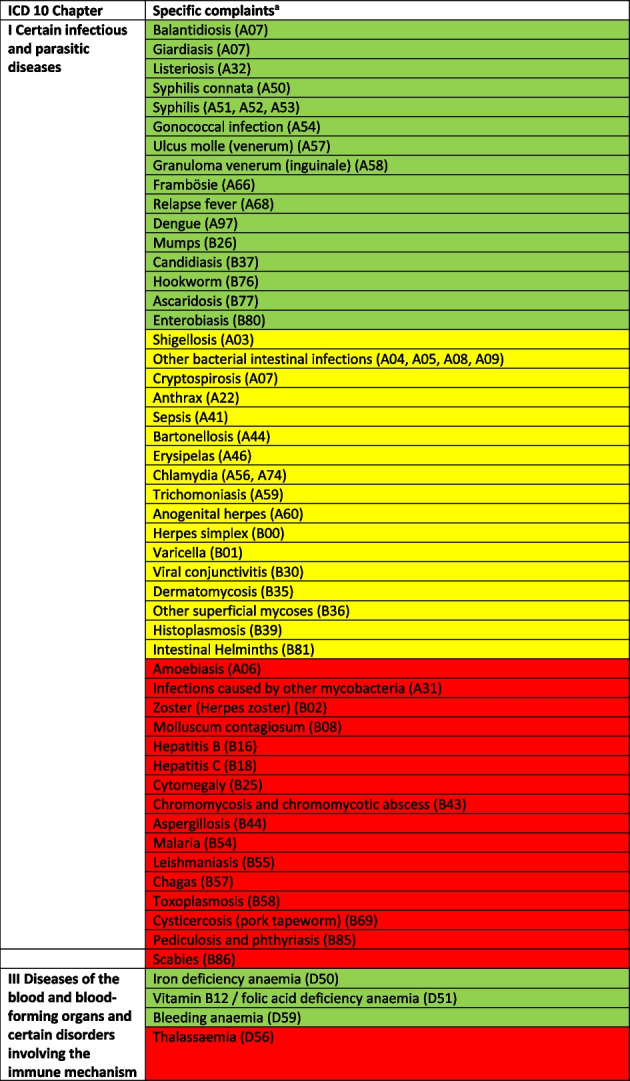

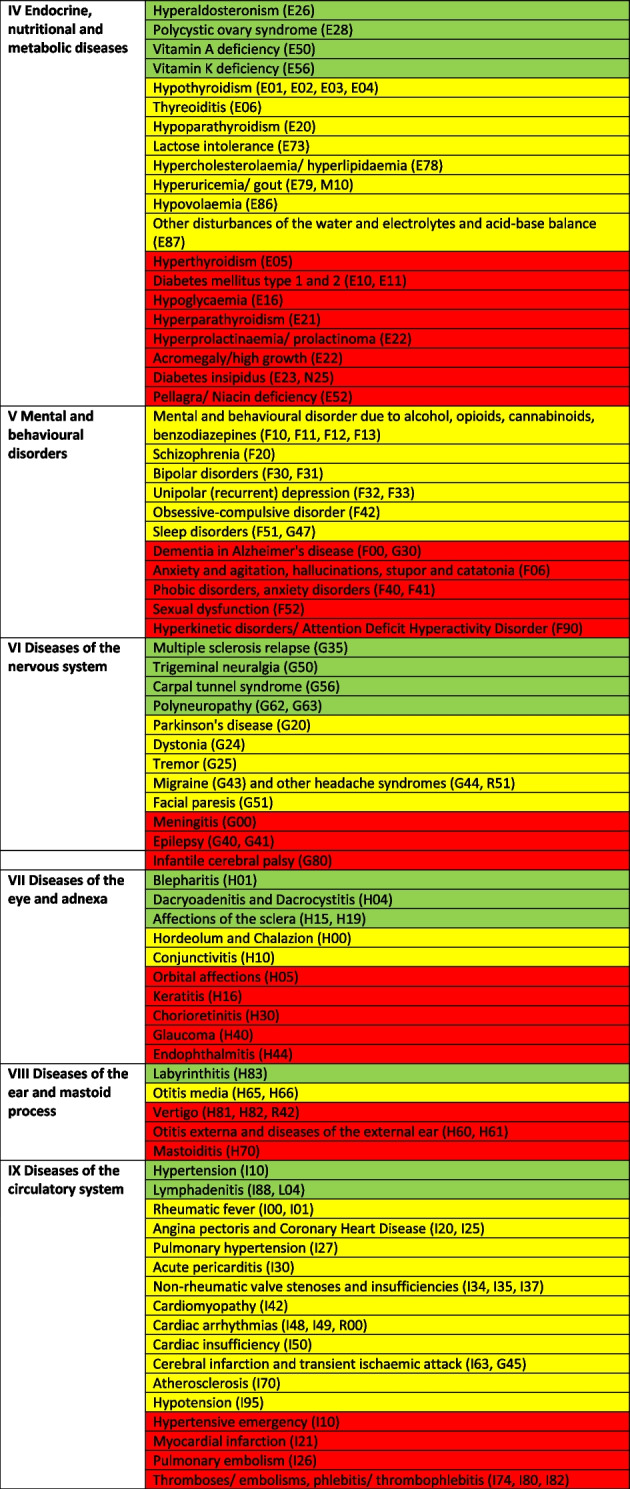

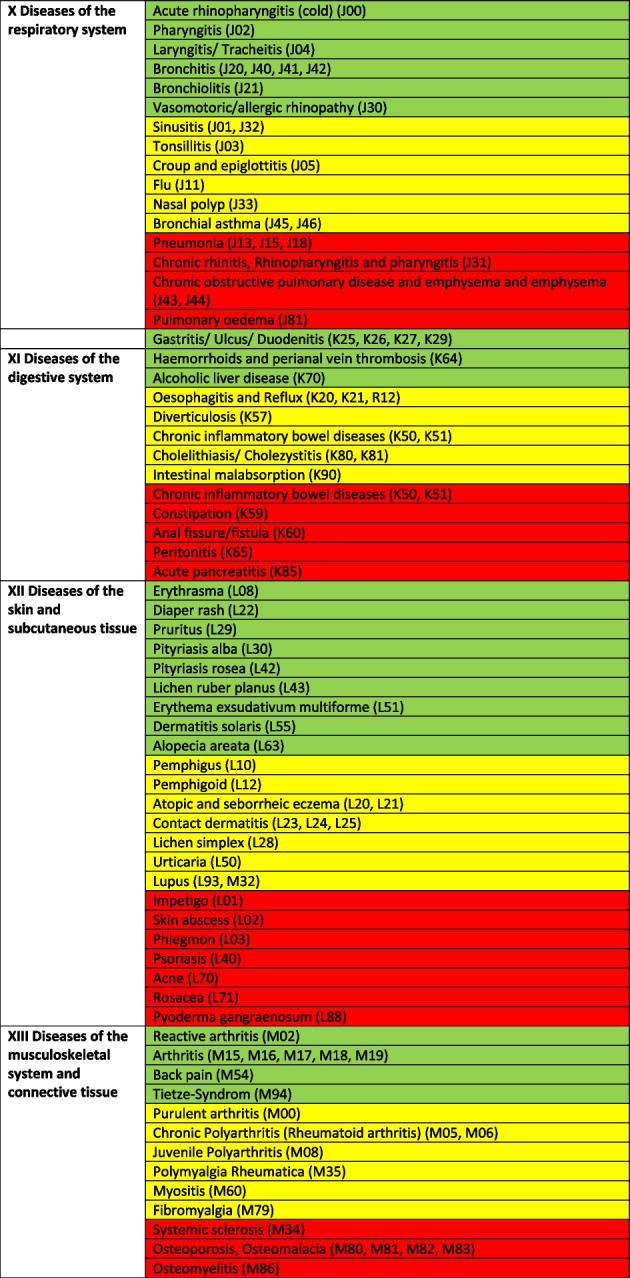

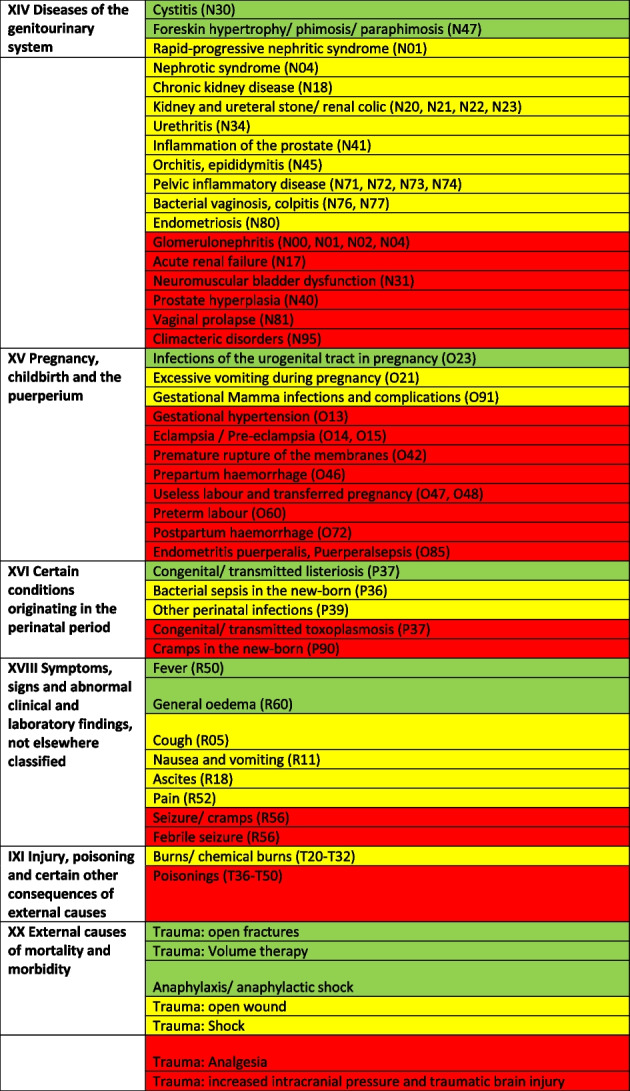
Overview about the diseases. Green: available medication sufficient to treat the diseases, yellow: available medication only partially sufficient to treat the diseases, red: available medication not sufficient to treat the diseases

^a^The categorization and information in the table is based on the sources as provided in the supplementary material 1

**Table 3:**
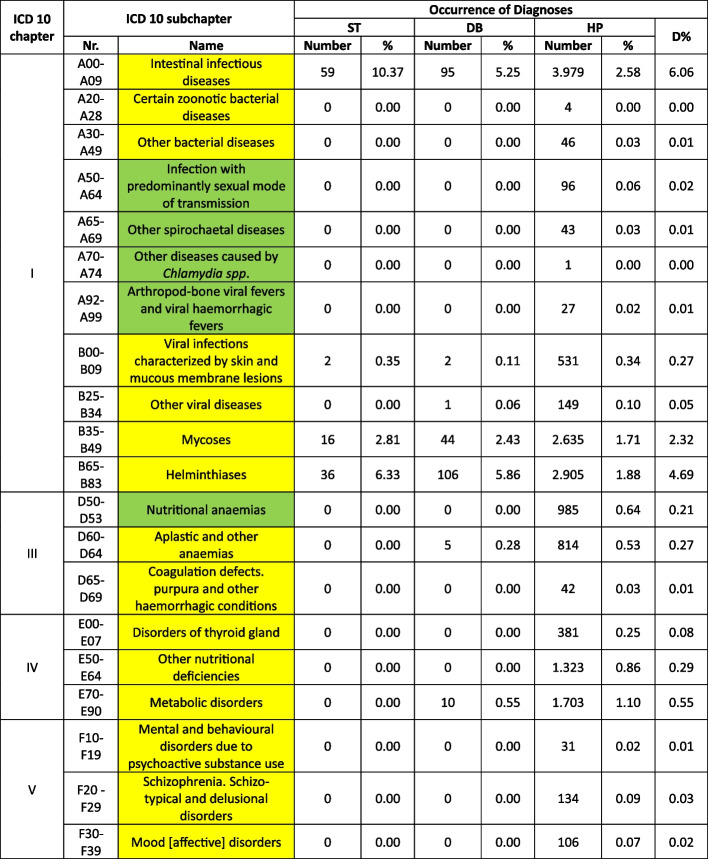

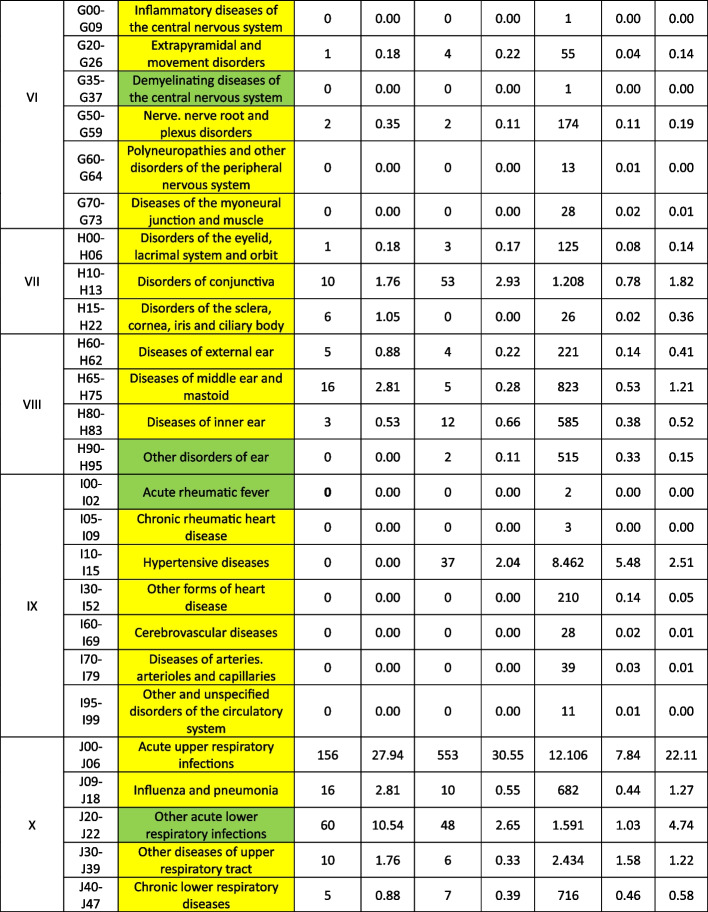

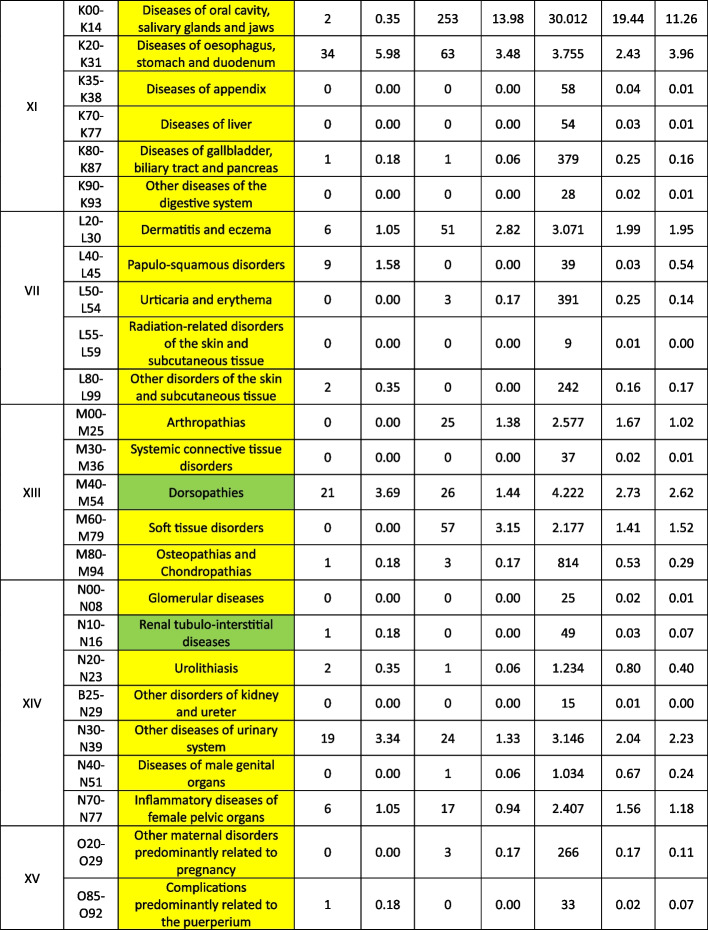

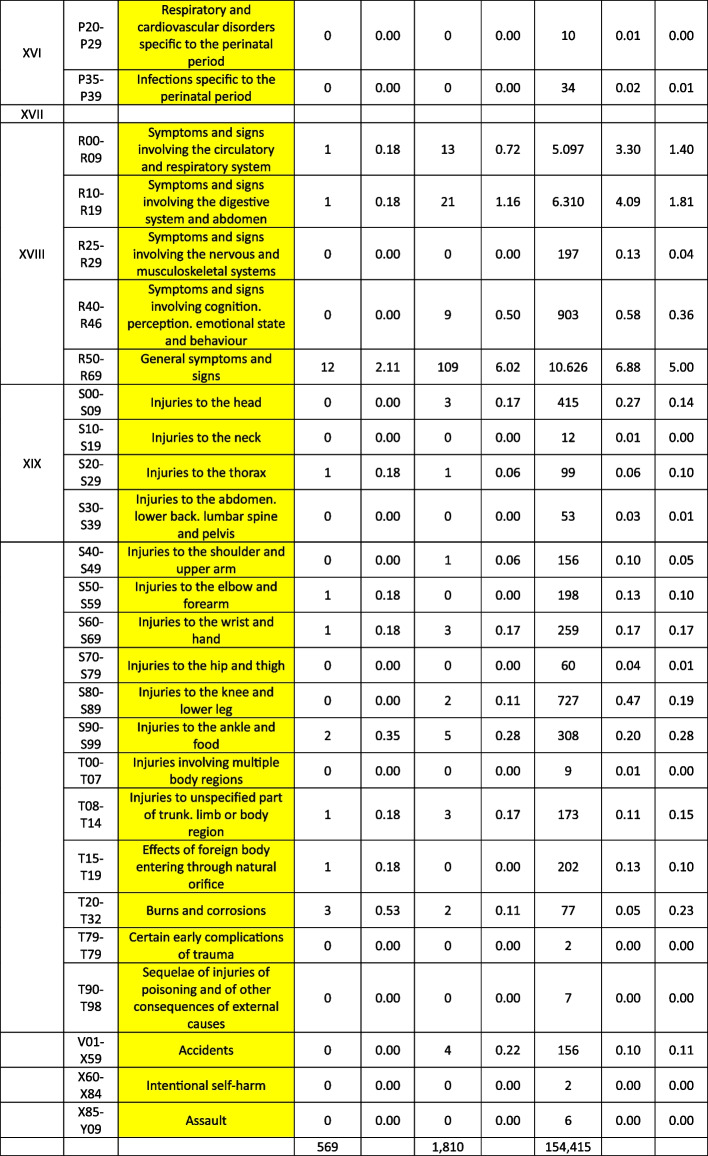
List of  and  diseases and occurrence of diagnoses in indigenous people in Sierra Nevada de Santa Marta, Colombia (ST = collected study data, DB = Dusakawi Health Brigades, HP = Dusakawi Health Points, see [2])

^a^The categorization and information in the table is based on the sources as provided in the supplementary Table 1

**Table 4:**
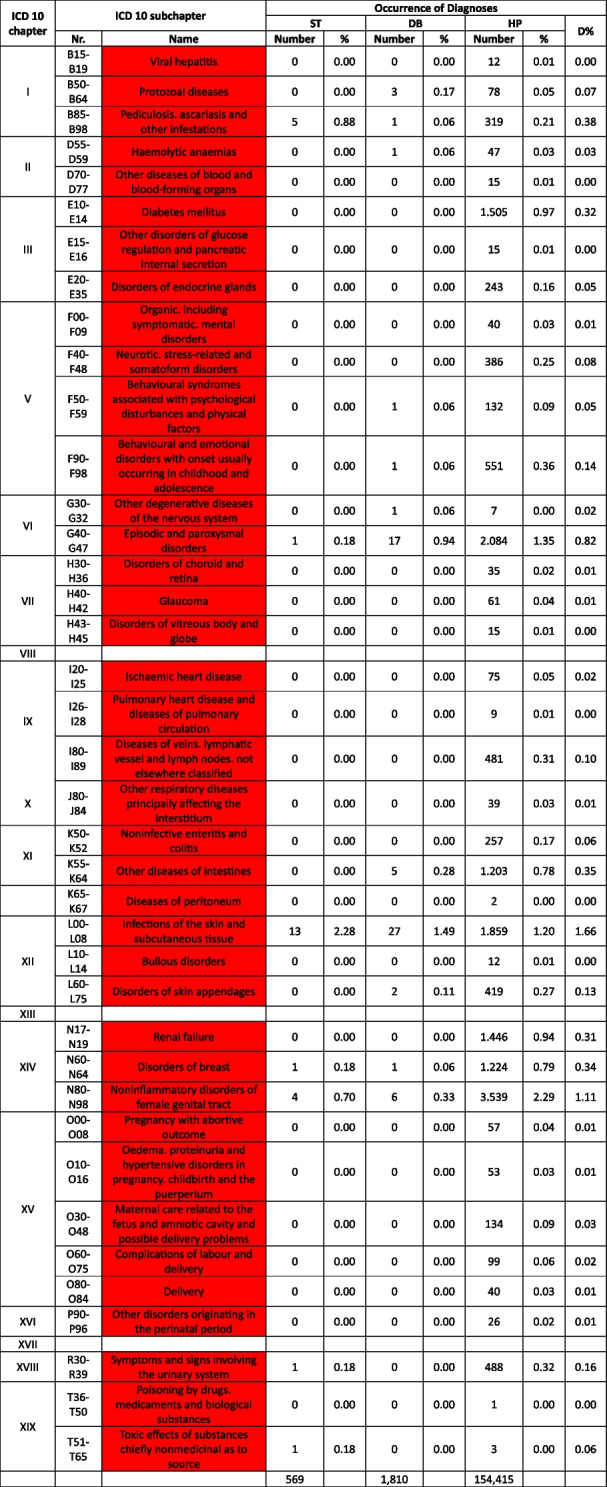
List of  diseases and occurrence of diagnoses in indigenous people in Sierra Nevada de Santa Marta, Colombia (ST = collected study data, DB = Dusakawi Health Brigades, HP = Dusakawi Health Points, see [2])

^a^The categorization and information in the table is based on the sources as provided in the supplementary Table 1

### Data management and statistical analysis

Documentation and linkage of information was performed within Excel 2016® (Microsoft Corp.). Within the three data sets, proportions of diagnoses per ICD-10 sub-chapter was calculated and an unweighted average proportion D% was calculated to rank the occurrence of diagnoses in the population of indigenous people.

## Results

### Composition of the dataset

During the on-site study in 2017–2018 as described previously [2], a total of 569 diagnoses was collected (ST dataset). Additional 1,810 diagnoses from 918 patients were provided by DB and 154,415 diagnoses were provided by HP. All three partial datasets were included in the analysis.

### In-depth analysis of the therapeutic options of the reported diseases

The Tables 2, 3 and 4 provide a comprehensive overview of the recorded diseases and the available therapeutic options. As described above, diagnoses that are covered completely with the available medication are marked in green, partially covered diagnoses in yellow and not at all covered ones in red. In total, only 12% of the diagnoses were considered as treatable according to good clinical practice, 56% as partially treatable and 32% as not sufficiently treatable at all. In the following, the focus is on more frequent infectious and non-infectious disease categories of which each provided more than 1% of total disease burden as well as their therapeutic coverage (see Tables 2, 3 and 4 for details).

Focusing on the infectious or at least partially infectious disease categories with an abundance of > 1% each, acute lower respiratory infections other than pneumonia and influenza (4.7%) were considered as treatable, acute upper respiratory infections (22.1%), diseases of the oral cavity, salivary glands and jaws (11.3%), intestinal infectious diseases (6.1%), general symptoms and signs (5.0%), helminthiases (4.7%), diseases of the esophagus, stomach and duodenum (4.0%), mycosis (3.2%), diseases of the urinary systems (2.2%), dermatitis and eczema (2.0%), disorders of the conjunctiva (1.8%), symptoms and signs involving the digestive system and abdomen (1.8%), influenza and pneumonia (1.7%), soft tissue disorders (1.5%), symptoms and signs involving the circulatory and respiratory system (1.4%), diseases of the middle ear and mastoid (1.2%), diseases of the upper respiratory tract other than acute infections (1.2%), inflammatory diseases of the female pelvic organs (1.2%) and arthropathias (1.0%) as partly/partially treatable and infectious disease of the skin and subcutaneous tissue (1.7%) as not adequately treatable.

From the non-infectious disease categories with an abundance of > 1% each, dorsopathias (2.6%) and hypertensive disease (2.5%) was considered as partly treatable and non-inflammatory disorders of the female genital tract (1.1%) as not adequately treatable. Other non-infectious disease categories were reported under the 1% threshold older as further assessed in the following.

### Neglect of disease categories

Diagnoses belonging to Chapter II (Neoplasms) and Chapter XVI (Congenital malformations, deformations and chromosomal abnormalities) of the ICD classification were only rarely found in all data provided. Also, complications during birth were rarely recorded and psychiatric disorders like depression were severely neglected. Details on the reported diseases are provided in Table 2.

## Discussion

The study was conducted to compare available medical treatment options and the actual medical need in an Columbian indigenous population called Wiwa. Confirming a recently stated suspicions [2], common and mild to moderate diseases and infections, for which monotherapies with, e.g., penicillin derivates are sufficient, were covered well. As long as the infections are in an early stage, e.g., in case of bronchitis and as long as the causative pathogen has no specific requirements (e.g., due to antibiotic resistances), the treatment options can be described as safe and sufficient. If medical conditions become more complex, the diseases can be treated only initially and/or incomplete. Therefore, they can progress to severe courses with complications, sequelae or even result in death. In addition, often just symptoms are treated, as suspected diagnoses cannot be confirmed and/or differential diagnoses cannot be made. *E. histolytica* infections, which were shown to be common in a previous assessment [15], are just an example. In case of amoebiasis, the acute infection can be treated with metronidazole or tinidazole onsite, however, the gut decontamination with paromomycin is missing, which is necessary to eliminate cysts. This can lead to relapsing disease including abscess formation in the liver [17].

As soon as complications and/or severe diseases occur, the medication list showed missing first and second line therapy options. For example, a bronchitis, which does not respond to amoxicillin treatment, can expand to a pneumonia, potentially leading to death. Even simple betalactam-betalactamase inhibitor combinations like amoxicillin/clavulanic acid are not in place. It is noticeable that for many other partly life-threatening diseases, e.g., diabetes mellitus type I, no medication is available at all. Looking at emergencies, the situation is even worse. For example, a myocardial infarction cannot be treated with anticoagulation and i.v. antihypertensive medication is not available. For complications during delivery, no therapeutic option is in place that could regulate or control the situation.

Another study question was the likely neglect of diagnostic categories in the documentation, which could be confirmed for several categories to be discussed in the following. First, although birth complications often show a fatal outcome for the child and/or the mother as reported for other indigenous populations [18], these events were rarely documented for the Wiwas. In contrast to this, mistreatment during childbirth in Colombian individuals has been addressed a problem for Colombian indigenous people by Gleason and colleagues [19]. The likely reason for the neglect in the Wiwas is the missing physician or midwife and the missing registration at all. Thematically closely related is the ICD chapter on child deformations, delivery complications and related topics, which were not represented at well. As known from the personal experience of the authors, these topics have to be considered as very stigmatized in the Wiwa communities. Further, no male physician is allowed to participate in a child birth. Midwifes are not in place, just elderly women trying to help the younger ones, but without the necessary medical education. A stillborn child is not mentioned any more, a deformed child is neglected and often dies due to missing medical support. E.g., a child with a harelip that cannot drink will die as first, the operation will not be covered in most cases and second, the mother cannot solve the situation by herself.

Further, it has to be assumed that many diseases are not even recorded at all as, e.g., accidents and other occasions, where the patient is not able to walk to the next far away health point or hospital, are not registered. This is also true for many chronically ill patients or elderly ones who cannot walk the necessary distance. This has also to be considered for severe ill persons, weak ones (e.g., individuals in need of a blood transfusion), women with birth complications, as well as weak or immature newborns.

Some ICD chapters are very neglected in the datasets at all. Cancer, for example, is one of those. Of course, Colombian indigenous persons suffer from cancer as well [20], but to get a treatment is basically impossible for the Wiwas. This is true for two main reasons: Most indigenous people are poor and have no or just a very basic health insurance. This health insurance covers, e.g., an x-ray analysis but no chemotherapy. Even some surgical procedures are excluded (e.g., transplantations). Secondly, it is too cost-intense for them to attend necessary examinations, as there is no adequate infrastructure nearby and distances are too far. Also psychiatric disorders are barely mentioned. On one hand, this is related to the traditions of the indigenous population, as, for example, psychiatric diseases like depression are neglected and respective complaints are socially discouraged by the communities. On the other hand, this could also be a matter of lacking knowledge, as many psychiatric diseases are not known to be a disease (alcoholism, etc.) among the communities. The neglect of psychiatric disorders, as practiced by the Wiwas, has been reported for other indigenous populations from other parts of the world as well [21]. Also, there is an increasing body of evidence that more specific disease entities that ocular disease entities are insufficiently treated in South-American indigenous populations as confirmed by Colombian authors as well [22].

Taken all this together, previously reported high morbidity and mortality rates [2–14] find additional explanations. To improve the situation, many efforts are necessary, starting with the expansion of the medication list by, e.g., second and third line antibiotic drugs to increase therapeutic options for the quantitatively dominating infectious diseases. However, their appropriate use will also require the necessary expertise in place. In line with this, more medical staff is needed to permanently work on-site at the already existing health points. Health points should be expanded and health brigades should stay longer and/or come more often. In addition, better infrastructure is essential. Life situations need to improve, that would prevent many diseases, e.g., by providing clean water, sanitation and safe housing. Trainings should be offered to educate the Wiwa communities about infections sources and prevention.

The study has a number of limitations. The most important limitation is the degree of uncertainty and imprecision necessarily associated with such holistic approaches due to errors by chance during data assessment and management as well as by the fact that the data by the healthcare providers were not specifically collected for the here-assessed study purpose. Second, the analyzed composite datasets did not discriminate between individuals claiming more than one complaint and individuals with just a single one, as only the number of diagnoses was recorded. Finally, funding constraints just allowing the financing of only a single study physician limited the time spans of medical on-site assessments in the included villages and thus the amount of data collected in this well-controlled way.

## Conclusions

Only mild to moderate symptoms and diseases can be therapeutically addressed in a sufficient way. For emergencies, complications and severe diseases, even basic medication is missing. Some ICD chapters like psychiatric diseases, birth-related complications and cancer are neglected in the list of available diagnoses. Awareness needs to be created for these shortcomings and the list of basic drugs has to be revised.

### Supplementary Information

Supplementary Material 1. Comprehensive overview of the references consulted to decide on therapeutical appropriateness.

Supplementary Material 2. Medication list provided by Dusakawi.

## Data Availability

No datasets were generated or analysed during the current study.
