# Supplementary material for: In-depth analysis of the medical supply for indigenous people in North-Eastern Colombia: a dominance of infectious diseases and only insufficient therapeutic options
Source: Arch Public Health. 2024 Jul 31;82:115. doi: 10.1186/s13690-024-01338-w (PMC11290115; doi:10.1186/s13690-024-01338-w)
Supplement: Supplementary file 1 — Supplementary Material 1. Comprehensive overview of the references consulted to decide on therapeutical appropriateness. [file 13690_2024_1338_MOESM1_ESM.docx]

**Supplementary material 1:** Comprehensive overview of the references consulted to decide on therapeutical appropriateness.

1. Bauer, H., et al., *Prevalence of Common Diseases in Indigenous People in Colombia.* Tropical Medicine and Infectious Disease, 2022. **7**(6): p. 109.

2. Herold, G. and Mitarbeiter, *Innere Medizin*. 2019: Herold, Gerd.

3. Krautzig, S., *Niere*, in *Basislehrbuch Innere Medizin*, H. Polster-Renz and S. Kautzig, Editors. 2013, Urban % Fischer: München. p. 801-873.

4. Leifeld, L., et al., *S2k Leitlinie Divertikelkrankheit/ Divertikulitis*. 2013, Deutschen Gesellschaft für Gastroenterologie, Verdauungs- und Stoffwechselkrankheiten (DGVS), Deutsche Gesellschaft für Allgemein- und Viszeralchirurgie (DGAV).

5. Layer, P., et al., *S3 Leitlinie Reizdarmsyndrom: Definition, Pathophysiologie, Diagnostik und Therapie.* Zeitschrift für Gastroenterologie, 2011. **49**: p. 237 – 293.

6. Gutt, C., et al., *Aktualisierte S3-Leitlinie der Deutschen Gesellschaft für Gastroenterologie, Verdauungs- und Stoffwechselkrankheiten (DGVS) und der Deutschen Gesellschaft für Allgemein- und Viszeralchirurgie (DGAV) zur Prävention, Diagnostik und Behandlung von Gallensteinen.* Zeitschrift für Gastroenterologie, 2018. **56**: p. 912–966.

7. Deutsche Gesellschaft für Gastroenterologie, V.-u.S.e.V.D., *S2k-Leitlinie Zöliakie*. 2014, Deutsche Gesellschaft für Gastroenterologie, Verdauungs- und Stoffwechselkrankheiten e.V. (DGVS): AWMF online.

8. Kardos, P., et al. *S2k-Leitlinie der Deutschen Gesellschaft für Pneumologie und Beatmungsmedizin zur Diagnostik und Therapie von erwachsenen Patienten mit Husten*. 2019.

9. *Leitlinie Palliativpflege: Übelkeit und Erbrechen*. 2014, Sektion Pflege der Deutschen Gesellschaft für Palliativmedizin (DGP).

10. Gerbes, A.R., et al. *Aktualisierung der S2k-Leitlinie der Deutschen Gesellschaft für Gastroenterologie, Verdauungs- und Stoffwechselkrankheiten (DGVS) „Komplikationen der Leberzirrhose“*. 2018.

11. Gerbes, A.L., et al., *S3 Leitlinie "Aszites, spontan bakterielle Peritonitis, hepatorenales Syndrom".* Zeitschrift für Gastroenterologie, 2011. **49**: p. 749 – 779.

12. Becker, A., M. Bercker, and P. Engeser, *Chronischer Schmerz*. 2013, Deutsche Gesellschaft für Allgemeinmedizin und Familienmedizin e.V. (DEGAM).

13. *Antibiotikatherapie bei HNO-Infektionen*. 2019, Deutsche Gesellschaft für Hals-Nasen-Ohren-Heilkunde, Kopf- und Hals-Chirurgie e.V. (DGHNO-KHC): AWMF online.

14. Damberg, A. and S. Denzinger. *Akute Epiglottitis*. 2020; Available from: <https://deximed.de/home/b/paediatrie/krankheiten/infektionen/epiglottitis-akute/#therapie>.

15. Issing, P.R., *Entzündliche Erkrankungen*, in *Facharztwissen HNO-Heilkunde*, M. Reiß, Editor. 2009, Springer Medizin Verlag: Heidelberg. p. 581-591.

16. *Influenza (Teil 1): Erkrankungen durch saisonale Influenzaviren*. RKI-Ratgeber 2018 19.01.2018 [cited 2020 02.06.2020]; Available from: [www.rki.de/ratgeber](file:///C:\Users\110442\Documents\G_lokal\Paper_Bauer_morbidity%20II\www.rki.de\ratgeber).

17. Lenz, M., I. Løge, and S.H. Glad Nordhahl. *Nasenpolypen*. 2020 [cited 2021 04.01.2021]; Available from: <https://deximed.de/home/klinische-themen/hals-nase-ohren/krankheiten/nase-und-nebenhoehlen/nasenpolypen/>.

18. *Nationale VersorgungsLeitlinie Asthma – Langfassung*. 2019, Bundesärztekammer (BÄK), Kassenärztliche Bundesvereinigung (KBV), Arbeitsgemeinschaft der Wissenschaftli-chen Medizinischen Fachgesellschaften (AWMF).

19. Risler, T., *Rapid progressive Glomerulonephritis*, in *Facharzt Nephrologie*, T. Risler, Editor. 2008, Elsevier GmbH: München. p. 285-287.

20. Weckmann, G., J.-F. Chenot, and S. Stracke, *Versorgung von Patienten mit chronischer nicht-dialysepflichtiger Nierenerkrankung in der Hausarztpraxis*, in *DEGAM-Leitlinie Nr. 22*. 2019, Deutsche Gesellschaft für Allgemeinmedizin und Familienmedizin (DEGAM): Berlin.

21. *S2k-Leitlinie zur Diagnostik, Therapie und Metaphylaxe der Urolithiasis - Aktualisierung 2018*. 2018, Arbeitskreis Harnsteine der Akademie der Deutschen Urologen, Deutsche Gesellschaft für Urologie e. V., Deutsche Gesellschaft für Urologie e. V.: AWMF online.

22. Bonkat, G., et al., *EAU Guidelines on Urological Infections*. 2019, European Association of Urology (EAU) Urological Infections Guidelines

23. Jung, N., S. Rieg, and C. Lehmann, *Klinikleitfaden Infektiologie*. Vol. 1. 2021: Elsevier.

24. *Leitfaden STI-Therapie*. 2015, Deutsche STI-Gesellschaft (DSTIG) zur Förderung der Sexuellen Gesundheit.

25. *Infektionen mit Chlamydia trachomatis*. 2016, Deutsche STI-Gesellschaft e.V. (DSTIG) - Gesellschaft zur Förderung der Sexuellen Gesundheit, Arbeitsgemeinschaft der Wissenschaftlichen Medizinischen Fachgesellschaften e.V.

26. Haag, P., N. Hanhart, and M. Müller, *Gynäkologie und Urologie für Studium und Praxis*. 2016, Breisach am Rhein: Müller, Markus.

27. Bahle, H., et al. *Bakterielle Vaginose*. 2019 [cited 2020 09.08.2020]; Available from: <https://deximed.de/home/b/gynaekologie/krankheiten/infektionen/bakterielle-vaginose/#autoren>.

28. Morshäuser, L., et al. *Endometriose*. 2020.

29. *Diagnostik und Therapie der Endometriose*. 2020, Deutsche Gesellschaft für Gynäkologie und Geburtshilfe e. V. (DGGG), Österreichische Gesellschaft für Gynäkologie und Geburtshilfe e.V. (ÖGGG), Schweizerische Gesellschaft für Gynäkologie und Geburtshilfe e.V. (SGGG), Arbeitsgemeinschaft der Wissenschaftlichen Medizinischen Fachgesellschaften e.V.: AWMF online.

30. *S2k-Leitlinie akute infektiöse Gastroenteritis im Säuglings-, Kindes- und Jugendalter*. 2019, Gesellschaft für Pädiatrische Gastroenterologie und Ernährung (GPGE), Arbeitsgemeinschaft der Wissenschaftlichen Medizinischen Fachgesellschaften e.V.

31. *Shigellose*. RKI-Ratgeber 2012 01.05.2012 [cited 2021 05.05.2021]; Available from: [www.rki.de/ratgeber](file:///C:\Users\110442\Documents\G_lokal\Paper_Bauer_morbidity%20II\www.rki.de\ratgeber).

32. Hagel, S., et al., *S2k-Leitlinie Gastrointestinale Infektionen und Morbus Whipple*. 2015, Deutsche Gesellschaft für Gastroenterologie, Verdauungs- und Stoffwechselkrankheiten e.V. (DGVS).

33. *Kryptosporidiose*, in *RKI Ratgeber*. 2019, Robert Koch Institut.

34. Ignatius, R. and G.-D. Burchard, *Protozoen*, in *Medizinische Mikrobiologie und Infektiologie*, S. Suerbaum, et al., Editors. 2020, Springer-Verlag GmbH Deutschland: Berlin, Heidelberg. p. 865-893.

35. *Anthrax in humans and animals*. 2008, World Health Organization, World Health Organization for Animal Health Food and Agriculture Organization of the United Nations: Geneva.

36. *S3-Leitlinie Sepsis–Prävention, Diagnose, Therapie und Nachsorge*. 2018, Deutsche Sepsis-Gesellschaft e.V. (DSG), Arbeitsgemeinschaft der Wissenschaftlichen Medizinischen Fachgesellschaften e.V. (AWMF).

37. *S2k Leitlinie Kalkulierte parenterale Initialtherapie bakterieller Erkrankungen bei Erwachsenen – Update 2018*. 2018, Paul-Ehrlich-Gesellschaft für Chemotherapie e.V. (PEG), Arbeitsgemeinschaft der Wissenschaftlichen Medizinischen Fachgesellschaften e.V.

38. Besier, M.S. and V.A.J. Kempf, *Bartonellen*, in *Medizinische Mikrobiologie und Infektiologie*, S. Suerbaum, et al., Editors. 2020, Springer-Verlag GmbH: Berlin, Heidelberg. p. 519-527.

39. Schlüter, D., *Trichomonaden*, in *Duale Reihe Medizinische Mikrobiologie*, H. Hof, D. Schlüter, and R. Dörries, Editors. 2019, Georg Thieme Verlag.

40. *WHO Guidelines for the Treatment of Genital Herpes Simplex Virus*. 2016, Worl Health Organisation.

41. Wollenberg, A. and H.-C. Rerinck, *Eczema herpeticatum.* Akt Dermatol, 2009. **35**: p. 319 – 321.

42. Schuster, V. and H.W. Kreth, *Varicella-Zoster-Virus-Infektionen*, in *Pädiatrie*, C.P. Speer, M. Gahr, and J. Dötsch, Editors. 2019, Springer-Verlag GmbH Deutschland: Berlin. p. 291-293.

43. Grehn, F., *Bindehaut*, in *Augenheilkunde*. 2019, Springer-Verlag GmbH Deutschland: Berlin. p. 111-139.

44. *Adenovirus-Konjunktivitis*. RKI-Ratgeber 2010 18.03.2010 [cited 2020 02.06.2020]; Available from: [www.rki.de/ratgeber](file:///C:\Users\110442\Documents\G_lokal\Paper_Bauer_morbidity%20II\www.rki.de\ratgeber).

45. *Tinea capitis*, in *AWMF-S1-Leitlinie*. 2019, Deutsche Dermatologische Gesellschaft e.V. (DDG), Deutsche Gesellschaft für Kinder- und Jugendmedizin e.V.: AWMF online.

46. Hofmann, H., *Mykosen der Haut*, in *Duale Reihe Dermatologie*, I. Moll, Editor. 2016, Georg Thieme Verlag.

47. Mayser, P., *Mykosen*, in *Braun-Falco’s Dermatologie, Venerologie und Allergologie*, G. Plewig, et al., Editors. 2018, Springer Berlin Heidelberg: Berlin, Heidelberg. p. 261-297.

48. *Histoplasmose*. Infektionskrankheiten A-Z 2020; Available from: <https://www.rki.de/DE/Content/InfAZ/P/Pilzinfektionen/Histoplasmose.html#doc2382370bodyText7>.

49. Schlüter, D., *Helminthen*, in *Duale Reihe Medizinische Mikrobiologie*, H. Hof, D. Schlüter, and R. Dörries, Editors. 2019, Georg Thieme Verlag.

50. *S2e-Leitline: Therapie der rheumatoiden Arthritis mit krankheitsmodifizierenden Medikamenten*. 2018, Deutsche Gesellschaft für Rheumatologie e.V. (DGRh), Arbeitsgemeinschaft der Wissenschaftlichen Medizinischen Fachgesellschaften e.V.

51. *S2k-Leitlinie „Therapie der Juvenilen Idiopathischen Arthritis“*. 2019, Deutsche Gesellschaft für Kinder- und Jugendmedizin e.V. (DGKJ, Gesellschaft für Kinder- und Jugendrheumatologie e.V. (GKJR, Arbeitsgemeinschaft der Wissenschaftlichen Medizinischen Fachgesellschaften e.V.

52. *S3-Leitlinie zur Behandlung der Polymyalgia rheumatica*. 2017, Deutsche Gesellschaft für Rheumatologie e.V. (DGRh), Arbeitsgemeinschaft der Wissenschaftlichen Medizinischen Fachgesellschaften e.V.

53. Wiendl, H., *Myositissyndrome*. 2014, Komission Leitlinien der Deutschen Gesellschaft für Neurologie.

54. *Definition, Pathophysiologie, Diagnostik und Therapie des Fibromyalgiesyndroms*. 2017, Deutsche Schmerzgesellschaft (DGSS): AWMF online.

55. Zealand, H.F.o.N. *New Zealand Guidelines for Rheumatic Fever: Diagnosis, Management and Secondary Prevention of Acute Rheumatic Fever and Rheumatic Heart Disease*. 2014.

56. *Nationale VersorgungsLeitlinie Chronische KHK – Langfassung*. 2019, Bundesärztekammer (BÄK), Kassenärztliche Bundesvereinigung (KBV), Arbeitsgemeinschaft der Wissenschaftlichen Medizinischen Fachgesellschaften (AWMF).

57. *Diagnostik und Therapie der pulmonalen Hypertonie*. 2015, European Society of Cardiology (ESC), Deutsche Gesellschaft für Kardiologie (DGK).

58. *Perikarderkrankungen*. 2015, European Society of Cardiology (ESC), Deutsche Gesellschaft für Kardiologie (DGK), Börm Bruckmeier Verlag GmbH.

59. *Herzklappenerkrankungen*. 2012, European Society of Cardiology (ESC), Deutsche Gesellschaft für Kardiologie (DGK), European Association for Cardio-Thoracic Surgery (EACTS).

60. *Diagnose und Behandlung der hypertrophen Kardiomyopathie*. 2015, European Society of Cardiology (ESC), Deutsche Gesellschaft für Kardiologie (DGK).

61. *Leitlinien für das Management von Vorhofflimmern*, in *ESC-Pocket-Guidelines*. 2012, Deutsche Gesellschaft für Kardiologie - Herz und Kreislaufforschung e.V., European Society of Cardiology.

62. *Ventrikuläre Arrythmien und Prävention des plötzlichen Herztodes*. 2015, Deutsche Gesellschaft für Kardiologie, European Society of Cardiology.

63. Monsieurs, K.G., et al., *Reanimation*, in *Leitlinien Kompakt*. 2015, Deutscher Rat für Wiederbelebung - German Resuscitation Council e.V.: Ulm.

64. *Sekundärprophylaxe ischämischer Schlaganfall und transitorische ischämische Attacke*. 2015, Deutsche Gesellschaft für Neurologie e.V. (DGN), Deutsche Schlaganfall-Gesellschaft e.V. (DSG).

65. Mader, F.M. and R. Schwenke, *Schlaganfall*, in *S3 Leitlinie*. 2020, Deutsche Gesellschaft für Allgemeinmedizin und Familienmedizin (DEGAM): Berlin.

66. Ludt, S., et al., *S3 Leitlinie Hausärztliche Risikoberatung zur kardiovaskulären Prävention*, in *DEGAM-Leitlinie Nr. 19*. 2017, Herausgeber Deutsche Gesellschaft für Allgemeinmedizin und Familienmedizin (DEGAM): Berlin.

67. Lawall, H., P. Huppert, and G. Rümenapf *S3-Leitlinie zur Diagnostik, Therapie und Nachsorge der peripheren arteriellen Verschlusskrankheit*. 2015.

68. *ESC Pocket Guidelines. Diagnose und Management von Synkopen*. 2018, Deutsche Gesellschaft für Kardiologie – Herz-und Kreislaufforschung e.V. , Börm Bruckmeier Verlag GmbH: Grünwald.

69. Schübel, J., et al., *Erhöhter TSH-Wert in der Hausarztpraxis*, in *DEGAM Leitlinie Nr. 18*. 2016, Deutsche Gesellschaft für Allgemeinmedizin und Familienmedizin (DEGAM): Berlin.

70. Bätge, B., C. Dodt, and H. Renz-Polster, *Endokrines System*, in *Basislehrbuch Innere Medizin*, H. Renz-Polster and S. Kautzig, Editors. 2013, Urban & Fischer: München. p. 671-729.

71. *S1 Leitlinie Hypoparathyreoidismus*, in *Leitlinie der Deutschen Gesellschaft für Kinderendokrinologie und diabetologie (DGKED) e.V.* 2016, Deutsche Gesellschaft für Kinderendokrinologie und –diabetologie (DGKED) e.V.: AWMF online.

72. Mach, F., et al., *Guidelines for the management of dyslipidaemias: lipid modification to reduce cardiovascular risk.* European Heart Journal, 2019. **41**: p. 111-188.

73. Prautzsch, H. and B. Engel, *Häufige Gichtanfälle und chronische Gicht*, in *DEGAM Leitlinie Nr 23*. 2019, Deutsche Gesellschaft für Allgemeinmedizin und Familienmedizin (DEGAM): Berlin.

74. *AWMF-S2k-Leitlinie „Diagnostik und Therapie des Pemphigus vulgaris / foliaceus und des bullösen Pemphigoids“*. 2019, Deutsche Dermatologische Gesellschaft e.V. (DDG).

75. *Leitlinie Neurodermitis [atopisches Ekzem; atopische Dermatitis]*. 2015, Deutsche Dermatologische Gesellschaft e.V. (DDG, Arbeitsgemeinschaft der Wissenschaftlichen Medizinischen Fachgesellschaften e.V.

76. Coors, E., *Seborrhoisches Ekzem*, in *Duale Reihe Dermatologie*, I. Moll, Editor. 2016, Georg Thieme Verlag.

77. Clark, G.W., S.M. Pope, and K.A. Jaboori, *Diagnosis and Treatment of Seborrheic Dermatitis.* American Family Physician, 2015. **91**: p. 185-190.

78. Brasch, J., et al., *Guideline contact dermatitis. S1-Guideline of the German Contact Allergy Group (DKG) of the German Dermatology Society (DDG), the Information Network of Dermatological Clinics (IVDK), the German Society for Allergology and Clinical Immunology (DGAKI), the Working Group for Occupational and Environmental Dermatology (ABD) of the DDG, the Medical Association of German Allergologists (AeDA), the Professional Association of German Dermatologists (BVDD) and the DDG.* Allergo J Int, 2014: p. 126–38.

79. Ständer, S., *Pruritus und Prurigo*, in *Braun-Falco’s Dermatologie, Venerologie und Allergologie*, G. Plewig, et al., Editors. 2018, Springer Berlin Heidelberg: Berlin, Heidelberg. p. 579-596.

80. *S2k-Leitlinie „Diagnostik und Therapie des kutanen Lupus erythematodes“*. 2020, Deutsche Dermatologische Gesellschaft e.V. (DDG): AWMF online.

81. Handke, M. *Systemischer Lupus erythemtodes (SLE)*. 2020; Available from: <https://deximed.de/home/b/rheumatologie/krankheiten/systemische-rheumatologische-erkrankungen/lupus-erythematodes-sle/#therapie>.

82. Fanouriakis, A., et al., *2019 update of the EULAR recommendations for the management of systemic lupus erythematosus.* 2019.

83. *Leitlinie Nr. 10 Hordeolum / Chalazion*. 2011, Berufsverband der Augenärzte Deutschlands e.V., Deutsche Ophthalmologische Gesellschaft e.V.

84. Quinn, C.J., et al., *Otometric Clinical Practice Guideline*, in *Care of the Patients with conjunctivitis*. 2002, American Optometric Association: St. Louis.

85. *Behandlung thermischer Verletzungen des Erwachsenen*. 2018, Deutsche Gesellschaft für Verbrennungsmedizin e.V. (DGV, Arbeitsgemeinschaft der Wissenschaftlichen Medizinischen Fachgesellschaften e.V. (AWMF).

86. *Leitlinie "zur Behandlung thermischer Verletzungen im Kindesalter (Verbrennung, Verbrühung)"*. 2015, Deutsche Gesellschaft für Kinderchirurgie, Arbeitsgemeinschaft der Wissenschaftlichen Medizinischen Fachgesellschaften e.V.: AWMF online.

87. *Idiopathisches Parkinson-Syndrom*. 2016, Deutsche Gesellschaft für Neurologie e.V. (DGN), Arbeitsgemeinschaft der Wissenschaftlichen Medizinischen Fachgesellschaften e.V. (AWMF).

88. Deuschl, G., *Tremor*. 2012, Deutsche Gesellschaft für Neurologie.

89. Diener H.-C., G.C., Kropp P. et al.,, *Therapie der Migräneattacke und Prophylaxe der Migräne, S1-Leitlinie.* Leitlinien für Diagnostik und Therapie in der Neurologie, 2018.

90. May, A. *Clusterkopfschmerz und trigeminoautonome Kopfschmerzen*. 2015.

91. Diener H.-C., G.C., Kropp P. et al.,, *Kopfschmerz bei Übergebrauch von Schmerz- oder Migränemitteln (Medication Overuse Headache = MOH)*, in *S1-Leitlinie*. 2018, Deutsche Gesellschaft für Neurologie (Hrsg.).

92. Wick, A. and T. Rizos, *Kopfschmerzen und Gesichtsneuralgien*, in *Neurologie*, W. Hacke, Editor. 2019, Springer-Verlag Berlin Heidelberg. p. 443-463.

93. al., H.J.G.e., *S2k-Leitlinie Therapie der idiopathischen Fazialisparese (Bell’s palsy).* Deutsche Gesellschaft für Neurologie, 2017.

94. Maschke, M. *Alkoholdelir und Verwirrtheitszustände*. 2014.

95. Pajonk, F.-G., T. Messer, and H. Berzewski *S2k-Leitlinie "Notfallpsychiatrie"*. 2019.

96. Ollenschläger, G. and O. Spigset. *Opioidvergiftung*. 2019 [cited 2020 03.08.2020]; Available from: <https://deximed.de/home/klinische-themen/erste-hilfe-notfallmedizin/notfaelle/vergiftungen/opioidvergiftung/>.

97. Heim, T. *Opioid-Entzugssyndrom*. 2020 [cited 2021 03.05.2021]; Available from: <https://deximed.de/home/klinische-themen/psychische-stoerungen/krankheiten/alkohol/entzugssyndrom/#quellen>.

98. W., H., *2. Aktualisierung der S3 Leitlinie „Langzeitanwendungen von Opioiden bei chronischen nicht-tumorbedingten Schmerzen „LONTS“.* Der Schmerz 2020, 2020. **34**.

99. *Cannabis-bezogene Störungen*. 2004, Deutsche Gesellschaft für Suchtforschung und Suchttherapie (DG Sucht), Deutsche Gesellschaft für Psychiatrie, Psychotherapie und Nervenheilkunde (DGPPN), Arbeitsgemeinschaft der Wissenschaftlichen Medizinischen Fachgesellschaften (AWMF)

100. *S3-Leitlinie zur Diagnostik und Therapie Bipolarer Störungen. Langversion, 2019*. 2019, Deutsche Gesellschaft für Bipolare Störungen e.V., Deutsche Gesellschaft für Psychiatrie und Psychotherapie, Psychosomatik, und Nervenheilkunde e.V.: AWMF online.

101. NVL-Programm von BÄK, K., AWMF, *S3-Leitlinie/Nationale VersorgungsLeitlinie Unipolare Depression – Kurzfassung*. 2017, DGPPN, BÄK, KBV, AWMF für die Leitliniengruppe Unipolare Depression.

102. Kordon, A., et al., *S3-Leitlinie Zwangsstörungen*, in *Kurzversion*. 2013, Deutsche Gesellschaft für Psychiatrie und Psychotherapie, Psychosomatik und Nervenheilkunde (DGPPN): AWMF online.

103. *Nichtorganische Schlafstörungen (F51)*. 2018, Deutsche Gesellschaft für Kinder- und Jugendpsychiatrie, Psychosomatik und Psychotherapie e.V. (DGKJP), Arbeitsgemeinschaft der Wissenschaftlichen Medizinischen Fachgesellschaften e.V.: AWMF online.

104. *DEGAM-Leitlinie Nr. 17*, in *Akuter Schwindel in der Hausarztpraxis*. 2015, Deutsche Gesellschaft für Allgemeinmedizin und Familienmedizin e.V. (DEGAM): Berlin.

105. Trifyllis, J. and I. Løge. *Hyperemesis gravidarum*. 2019 Available from: <https://deximed.de/home/b/schwangerschaft-geburtshilfe/schwangerschaft/beschwerden-in-der-schwangerschaft/hyperemesis-gravidarum/#autoren>.

106. Fillenberg, S. and L. Lasch, *Geburtshilfe*, in *Basiswissen Gynäkologie und Geburtshilfe*. 2017, Springer-Verlag GmbH Deutschland Berlin. p. 171-257.

107. Lasch, L., *Wochenbett*, in *Basiswissen Gynäkologie und Geburtshilfe*. 2017, Springer-Verlag GmbH Deutschland: Berlin. p. 259-266.

108. *Therapieempfehlungen für die Notfallmedizin*. 2019, Fortbildungsausschuss der Arbeitsgemeinschaft in Norddeutschland tätiger Notärzte e.V. (AGNN).

109. Niethard, F.-U., J. Pfeil, and P. Biberthaler, *Grundlagen der Unfallchirurgie*. 2017, Georg Thieme Verlag KG: Stuttgart. p. 292-356.

110. Herzig, N., et al. *Traumatische und chirurgische Wunden* 2017 [cited 2021 18.05.2021]; Available from: <https://deximed.de/home/klinische-themen/erste-hilfe-notfallmedizin/notfaelle/trauma-und-fraktur/wunden-traumatische-chirurgische/#therapie>.

111. *Bakterielle Infektionen beim Neugeborenen*. 2019, Gesellschaft für Neonatologie und pädiatrische Intensivmedizin e.V. (GNPI), Arbeitsgemeinschaft der Wissenschaftlichen Medizinischen Fachgesellschaften e.V.

112. *Prophylaxe der Neugeborenensepsis - frühe Form - durch Streptokokken der Gruppe B*. 2016, Gesellschaft für Neonatologie und pädiatrische Intensivmedizin e.V. (GNPI), Arbeitsgemeinschaft der Wissenschaftlichen Medizinischen Fachgesellschaften (AWMF).

113. Schmiemann, G., K. Gebhardt, and E. Hummers, *S3 Leitlinie: Brennen beim Wasserlassen*, in *DEGAM-Leitlinie Nr. 1*. 2018, Deutsche Gesellschaft für Allgemeinmedizin und Familienmedizin e.V.: AWMF online.

114. *Diagnostik und Therapie der Gonorrhoe*. 2018, Deutsche STI-Gesellschaft e.V. (DSTIG), Gesellschaft zur Förderung der Sexuellen Gesundheit, Arbeitsgemeinschaft der Wissenschaftlichen Medizinischen Fachgesellschaften e.V.

115. Deutsche Gesellschaft für Gastroenterologie, V.-u.S.e.V.D., *Aktualisierte S3-Leitlinie „Diagnostik und Therapie des M. Crohn“ 2014*. 2014, Arbeitsgemeinschaft der Wissenschaftlichen Medizinischen Fachgesellschaften (AWMF).

116. Kucharzik, T., et al. *Aktualisierte S3 Leitlinie Colitis ulcerosa*. 2019.

117. *S2k-Leitlinie Chronische Obstipation: Definition, Pathophysiologie, Diagnostik und Therapie*. 2013, Deutsche Gesellschaft für Gastroenterologie, Verdauungs- und Stoffwechselkrankheiten e.V. (DGVS), Deutsche Gesellschaft für Neurogastroenterologie und Motilität e.V. (DGNM, Arbeitsgemeinschaft der Wissenschaftlichen Medizinischen Fachgesellschaften e.V.

118. *Erweiterte S3-Leitlinie Palliativmedizin für Patienten mit einer nicht-heilbaren Krebserkrankung*, in *Langversion 2.1*. 2020, Leitlinienprogramm Onkologie, Deutsche Gesellschaft für Palliativmedizin (DGP): AWMF online.

119. Marti, L., et al., *S3 Leitlinie: Analfissur.* Coloproctology, 2020. **42**: p. 90-196.

120. Karsch-Völk, M., G. Ollenschläger, and F. Bærheim. *Akutmedizinische Behandlung von Schwerverletzten am Unfallort* 2017 [cited 2020 19.08.2020]; Available from: <https://deximed.de/home/klinische-themen/erste-hilfe-notfallmedizin/erste-hilfe/trauma-behandlung-am-unfallort/#transport>.

121. Ewig, S., et al., *Behandlung von erwachsenen Patienten mit ambulant erworbener Pneumonie und Prävention*. Update 2016, Deutsche Gesellschaft für Pneumologie und Beatmungsmedizin, PaulEhrlich-Gesellschaft für Chemotherapie, Deutsche Gesellschaft für Infektiologie, Kompetenznetzwerk CAPNETZ, Österreichische Gesellschaft für Pneumologie, der Österreichische Gesellschaft für Infektionskrankheiten und Tropenmedizin und der Schweizerische Gesellschaft für Pneumologie.

122. Vogelmeier, C., et al., *S2k-Leitlinie zur Diagnostik und Therapie von Patienten mit chronisch obstruktiver Bronchitis und Lungenemphysem (COPD)*. 2018, Deutschen Gesellschaft für Pneumologie und Beatmungsmedizin e.V. und Deutschen Atemwegsliga e.V. unter Beteiligung der Österreichischen Gesellschaft für Pneumologie.

123. Floege, A. and J. Floege, *KDIGO-Leitlinien zur Behandlung von Glomerulonephritiden.* Der Nephrologe, 2013. **4**.

124. Haensch, C.-A.e.a., *Diagnostik und Therapie von neurogenen Blasenstörungen, S1 Leitlinie.* Leitlinien für Diagnostik und Therapie in der Neurologie., 2020.

125. Höfner, K., et al., *Leitlinie zur Therapie des benignen Prostatasyndroms der Qualität S2e*. 2012, Arbeitskreis Benignes Prostatasyndrom (AK BPS) der Akademie der Deutschen Urologen, Deutsche Gesellschaft für Urologie e.V., Berufsverband der Deutschen Urologen e.V.: AWMF online.

126. Höfner, K., et al., *S2e Leitlinie der deutschen Urologen: Konservative und medikamentöse Therapie des benignen Prostatasyndroms.* Der Urologe, 2016. **55**: p. 184-194.

127. Baum, E., et al. *Descensus genitalis/Genitalprolaps*. 2019 27.05.2019 [cited 2020 07.08.2020]; Available from: <https://deximed.de/home/klinische-themen/gynaekologie/krankheiten/verschiedene-krankheiten/descensus-genitalis-genitalprolaps/#autoren>.

128. Heim, T.M. and E. Baum. *Klimakterium*. 2020 [cited 2020 17.09.2020]; Available from: <https://deximed.de/home/klinische-themen/gynaekologie/krankheiten/beratungsanlaesse/klimakterium/>.

129. *AWMF S2k-Leitlinie „Diagnostik und Therapie des Zoster und der Postzosterneuralgie“*. 2019, Deutsche Dermatologische Gesellschaft e.V. (DDG), Paul-Ehrlich-Gesellschaft für Chemotherapie e.V.: AWMF online.

130. Meissner, M., *Verrucae vulgares*, in *Duale Reihe Dermatologie*, I. Moll, Editor. 2016, Georg Thieme Verlag.

131. Lenz, M., et al. *Warzen an Fingern und Füßen*. 2018 [cited 2021 19.05.2021]; Available from: <https://deximed.de/home/klinische-themen/haut/krankheiten/papuloese-hauterkrankungen/warzen-an-fingern-und-fuessen/#autoren>.

132. Meissner, M., *Viruskrankheiten der Haut*, in *Duale Reihe Dermatologie*, I. Moll, Editor. 2016, Georg Thieme Verlag.

133. *Hepatitis B und D*. RKI-Ratgeber 2016 20.05.2016 02.06.2020]; Available from: [www.rki.de/ratgeber](file:///C:\Users\110442\Documents\G_lokal\Paper_Bauer_morbidity%20II\www.rki.de\ratgeber).

134. Deutsche Gesellschaft für Gastroenterologie, V.-u.S.e.V.D., *S3-Leitlinie der Deutschen Gesellschaft für Gastroenterologie, Verdauungs- und Stoffwechselkrankheiten (DGVS) zur Prophylaxe, Diagnostik und Therapie der Hepatitis-B-Virusinfektion*.

135. Sarrazin, C., et al., *S3-Leitlinie "Prophylaxe, Diagnostik und Therapie der Hepatitis-C-Virus (HBC) Infektion".* Zeitschrift für Gastroenterologie, 2018. **56**: p. 756–838.

136. *Zytomegalievirus-Infektion*. RKI-Ratgeber 2014 20.01.2014 [cited 2020 02.06.2020]; Available from: [www.rki.de/ratgeber](file:///C:\Users\110442\Documents\G_lokal\Paper_Bauer_morbidity%20II\www.rki.de\ratgeber).

137. Hof, H., *Schimmelpilze*, in *Duale Reihe Medizinische Mikrobiologie*, H. Hof, D. Schlüter, and R. Dörries, Editors. 2019, Georg Thieme Verlag.

138. *Diagnostik und Therapie der Malaria*. 2015, Deutsche Gesellschaft für Tropenmedizin und Internationale Gesundheit (DTG), Arbeitsgemeinschaft der Wissenschaftlichen Medizinischen Fachgesellschaften e.V.: AWMF online.

139. *Leitlinie: Diagnostik und Therapie der viszeralen Leishmaniasis (Kala-Azar)*. 2016, Deutsche Gesellschaft für Tropenmedizin, Reisemedizin und Globale Gesundheit e.V. (DTG), Arbeitsgemeinschaft der Wissenschaftlichen Medizinischen Fachgesellschaften e.V.

140. von Stebut, E., *Protozoonosen der Haut*, in *Braun-Falco’s Dermatologie, Venerologie und Allergologie*, G. Plewig, et al., Editors. 2018, Springer Berlin Heidelberg: Berlin, Heidelberg. p. 391-406.

141. *Chagas disease (American trypanosomiasis)*. Available from: <https://www.who.int/health-topics/chagas-disease#tab=tab_3>.

142. *RKI-Ratgeber Toxoplasmose.* Epidemiologisches Bulletin, 2018. **42**: p. 451-457.

143. Pearson, R.D. *Taenia solium (Schweinebandwurm)-Infektio und Zystizerkose*. 2018; Available from: <https://www.msdmanuals.com/de-de/profi/infektionskrankheiten/zestoden-bandw%C3%BCrmer/taenia-solium-schweinebandwurm-infektion-und-zystizerkose>.

144. *Kopflausbefall*. RKI-Ratgeber 2008 17.11.2008 [cited 2020 02.06.2020]; Available from: [www.rki.de/ratgeber](file:///C:\Users\110442\Documents\G_lokal\Paper_Bauer_morbidity%20II\www.rki.de\ratgeber).

145. Geginat, G., *Ordnung Anoplura (Läuse)*, in *Duale Reihe Medizinische Mikrobiologie*, H. Hof, D. Schlüter, and R. Dörries, Editors. 2019, Georg Thieme Verlag.

146. Specker, C. and T. Bitsch, *Kollagenosen*, in *Klinikleitfaden Rheumatologie*. 2018, Bitsch, Thomas, Elsevier GmbH Deutschland: München. p. 277-307.

147. *Prophylaxe, Diagnostik und Therapie der Osteoporose bei postmenopausalen Frauen und bei Männern*, in *Leitlinie des Dachverbands der Deutschsprachigen Wissenschaftlichen Osteologischen Gesellschaften e.V.* 2017, Arbeitsgemeinschaft der Wissenschaftlichen Medizinischen Fachgesellschaften e.V.: AWMF online.

148. *Akute und chronische exogene Osteomyelitis langer Röhrenknochen des Erwachsenen*. 2017, Deutsche Gesellschaft für Unfallchirurgie e.V. (DGU), Arbeitsgemeinschaft der Wissenschaftlichen Medizinischen Fachgesellschaften e.V.

149. *ESC Pocket Guidelines: Therapie des akuten Herzinfarktes bei Patienten mit ST-Streckenhebung* 2017, Deutsche Gesellschaft für Kardiologie, European Society of Cardiology.

150. Hach-Wunderle, V., et al., *Leitlinie zur Diagnostik und Therapie der Venenthrombose und der Lungenembolie*, ed. D.Ä. GmbH. 2017: Deutsche Gesellschaft für Angiologie-Gesellschaft für Gefäßmedizin e. V.

151. Encke, A., et al., *S3 Leitlinie: Prophylaxe der venösen Thrombembolie (VTE)*. 2015, Arbeitsgemeinschaft der Wissenschaftlichen Medizinischen Fachgesellschaften e.V.: AWMF online.

152. *Nationale VersorgungsLeitlinie Typ-2-Diabetes – Teilpublikation der Langfassung*. 2021, Bundesärztekammer (BÄK), Kassenärztliche Bundesvereinigung (KBV), Arbeitsgemeinschaft der Wissenschaftlichen Medizinischen Fachgesellschaften (AWMF).

153. *S3-Leitlinie Therapie des Typ-1-Diabetes*, in *Langfassung*. 2018, Deutsche Diabetes Gesellschaft e.V.: AWMF online.

154. Renz-Polster, H. and H.-J. Frercks, *Stoffwechsel*, in *Basislehrbuch Innere Medizin*, H. Renz-Polster and S. Krautzig, Editors. 2013, Elsevier GmbH: München. p. 731-799.

155. *Diabetes insipidus neurohormonalis (D. i. centralis)*. 2011, Deutsche Gesellschaft für Kinder- und Jugendmedizin e.V. (DGKJ), Arbeitsgemeinschaft der Wissenschaftlichen Medizinischen Fachgesellschaften e.V.

156. Montag, A., *Das Pellagra gestern und heute – Auf den Spuren eines Jahrhunder trätsels.* Aktuelle Dermatologie, 2016. **42**: p. 131-138.

157. Meissner, M., *Impetigo contagiosa*, in *Duale Reihe Dermatologie*, I. Moll, Editor. 2016, Georg Thieme Verlag.

158. Abeck, D., *Staphylokokken und Streptokokken*, in *Braun-Falco`s Dermatologie, Venerologie und Allergologie*, G. Plewig, et al., Editors. 2012, Springer-Verlag: Berlin Heidelberg. p. 143-169.

159. Meissner, M., *Follikulitis*, in *Duale Reihe Dermatologie*, I. Moll, Editor. 2016, Georg Thieme Verlag.

160. Nast, A., et al. *Therapie der Psoriasis vulgaris*. 2017.

161. Nast, A., et al., *Behandlung der Akne*. 2011, Deutsche Dermatologische Gesellschaft: AWMF online.

162. *Rosazea*, in *Leitlinie der Deutschen Dermatologischen Gesellschaft*. 2013, Deutsche Dermatologische Gesellschaft e.V. (DDG): AWMF online.

163. Kohrmeyer, K. and F.A. Bahmer, *Arteritiden*, in *Duale Reihe Dermatologie*, I. Moll, Editor. 2016, Georg Thieme Verlag.

164. *Leitlinie Nr 13: Keratitis*. 2011, Berufsverband der Augenärzte Deutschlands e.V., Deutsche Ophthalmologische Gesellschaft e.V.

165. Grehn, F., *Hornhaut*, in *Augenheilkunde*. 2019, Springer-Verlag GmbH Deutschland: Berlin. p. 141-188.

166. *ICO Guidelines for Glaucoma Eye Care*. 2015, International Council of Ophthalmology.

167. Müller, D. and H. Desel, *Ursachen, Diagnostik und Therapie häufiger Vergiftungen.* Dtsch Arztebl International, 2013. **110**(41): p. 690-700.

168. Pfister, H.-W., *S2k-Leitlinie Ambulant erworbene bakterielle (eitrige) Meningoenzephalitis im Erwachsenenalter*. 2015, Leitlinien der Deutschen Gesellschaft für Neurologie: Deutsche Gesellschaft für Neurologie.

169. Meyding-Lamadé, U., *Virale Meningoenzephalitis*, in *S1-Leitlinie*. 2018, Deutsche Gesellschaft für Neurologie.

170. Elger, C.E. and R. Berkenfeld, *S1 Leitlinie Erster epileptischer Anfall und Epilepsien im Erwachsenenalter.* Deutsche Gesellschaft für Neurologie, Hrsg. Leitlinien für Diagnostik und Therapie in der Neurologie, 2017.

171. Rosenow, F. *Status epilepticus im Erwachsenenalter*. 2012.

172. Ollenschläger, G., et al. *Zerebralparese (CP)*. 2019 [cited 2021 18.05.2021]; Available from: <https://deximed.de/home/klinische-themen/paediatrie/krankheiten/neurologie/zerebralparese-cp/#quellen>.

173. Deutsche Gesellschaft für Psychiatrie und Psychotherapie, P.u.N.D. and D.G.f.N. (DGN) *S3-Leitlinie "Demenzen"*. 2016.

174. Schulz, J.B., K. Hess, and A.C. Ludolph, *Kognitive Einschränkungen und Demenzen*, in *Neurologie*. 2019, Hacke, Werner, Springer-Verlag GmbH Deutschland: Heidelberg. p. 645-663.

175. *S3-Leitlinie Schizophrenie. Kurzfassung, 2019*. DGPPN e.V. (Hrsg.) für die Leitliniengruppe.

176. *S3-Leitlinie Behandlung von Angststörungen*. 2014, Deutsche Gesellschaft für Psychosomatische Medizin und Ärztliche Psychotherapie e.V. (DGPM), Arbeitsgemeinschaft der Wissenschaftlichen Medizinischen Fachgesellschaften e.V.

177. Gasser, T., *Sexualpathologie des Mannes*, in *Basiswissen Urologie*. 2019, Springer-Verlag GmbH: Berlin, Heidelberg. p. 107-114.

178. *Langfassung der interdisziplinären evidenz- und konsensbasierten (S3) Leitlinie "Aufmerksamkeitsdefizit-/ Hyperaktivitätsstörung (ADHS) im Kindes-, Jugend- und Erwachsenenalter"*. 2017, Deutsche Gesellschaft für Kinder- und Jugendpsychiatrie, Psychosomatik und Psychotherapie e.V. (DGKJP), Deutsche Gesellschaft für Psychiatrie und Psychotherapie, Psychosomatik und Nervenheilkunde e.V. (DGPPN), Deutsche Gesellschaft für Sozialpädiatrie und Jugendmedizin e.V. (DGSPJ), Arbeitsgemeinschaft der Wissenschaftlichen Medizinischen Fachgesellschaften e.V.

179. Piper, W., *Thalassämien*, in *Innere Medizin*, W. Piper, Editor. 2007, Springer Medizin Verlag: Heidelberg. p. 626-629.

180. *Hypertensive Schwangerschaftserkrankungen: Diagnostik und Therapie*. 2019, Deutsche Gesellschaft für Gynäkologie und Geburtshilfe e.V. (DGGG), Österreichische Gesellschaft für Gynäkologie und Geburtshilfe (OEGGG), Schweizerische Gesellschaft für Gynäkologie und Geburtshilfe (SGGG)Arbeitsgemeinschaft der Wissenschaftlichen , Medizinischen Fachgesellschaften e.V. (AWMF).

181. *Geburtseinleitung*. 2020, Deutsche Gesellschaft für Gynäkologie und Geburtshilfe e.V. (DGGG), Österreichische Gesellschaft für Gynäkologie und Geburtshilfe e.V. (ÖGGG), Schweizerische Gesellschaft für Gynäkologie und Geburtshilfe e.V. (SGGG), Arbeitsgemeinschaft der Wissenschaftlichen Medizinischen Fachgesellschaften e.V.

182. Kainer, F. and U. Hasbargen, *Notfälle in der Geburtshilfe – peripartale Blutungen.* Dtsch Arztebl International, 2008. **105**(37): p. 629-37.

183. *Leitlinie Prävention und Therapie der Frühgeburt*. 2020, Deutsche Gesellschaft für Gynäkologie und Geburtshilfe (DGGG), Österreichische Gesellschaft für Gynäkologie und Geburtshilfe (ÖGGG), Schweizerische Gesellschaft für Gynäkologie und Geburtshilfe (SGGG, Arbeitsgemeinschaft der Wissenschaftlichen Medizinischen Fachgesellschaften e.V.

184. Trifyllis, J., et al. *Postpartale Blutung*. 2019; Available from: <https://deximed.de/home/b/schwangerschaft-geburtshilfe/geburt-wochenbett/wochenbett/postpartale-blutung/#autoren>.

185. *Peripartale Blutungen, Diagnostik und Therapie*. 2016, Deutsche Gesellschaft für Gynäkologie und Geburtshilfe e.V. (DGGG), Österreichische Gesellschaft für Gynäkologie und Geburtshilfe (OEGGG), Schweizerische Gesellschaft für Gynäkologie und Geburtshilfe (SGGG), Arbeitsgemeinschaft der Wissenschaftlichen Medizinischen Fachgesellschaften e.V. (AWMF).

186. *Bacterial Sepsis following pregnancy*, in *Green-top Guideline No. 64b*. 2012, Royal College of Obstetricians & Gynaecologists.

187. *S3 – Leitlinie Polytrauma/ Schwerverletzten-Behandlung*. 2016, Deutsche Gesellschaft für Unfallchirurgie (federführend): Berlin.

188. Wentzell, R., *Neonatologie*, in *Klinikleitfaden Pädiatrie*, U.F.V. Elsevier GmbH, Editor. 2014, Illing, Stephan; Claßen, Martin: München.
