# Supplementary material for: In-depth analysis of the medical supply for indigenous people in North-Eastern Colombia: a dominance of infectious diseases and only insufficient therapeutic options
Source: Arch Public Health. 2024 Jul 31;82:115. doi: 10.1186/s13690-024-01338-w (PMC11290115; doi:10.1186/s13690-024-01338-w)
Supplement: Supplementary file 2 — Supplementary Material 2. Medication list provided by Dusakawi. [file 13690_2024_1338_MOESM2_ESM.docx]

**Supplementary material 1:** Detailed composition of the available medication.

| **Substance** | **Dose** | **Pharmaceutical form** |
| --- | --- | --- |
| Acetylsalicylic acid | 100 mg | Tablet |
| Acyclovir | 200 mg  5% 15 g | Tablet  Cream |
| Adrenaline | 1 mg | Vial |
| Albendazole | 100 mg/ 5 ml 20 ml  200 mg | Suspension  Tablet |
| Allopurinol | 100 mg,  300 mg | Tablet  Tablet |
| Alpha methyldopa | 250 mg | Tablet |
| Aluminium hydroxide + magnesium + Simeticone | 360 ml | Suspension |
| Amantadine | 100 mg | Capsule |
| Amikacin | 100 mg  500 mg | Vial  Vial |
| Aminophylline | 240 mg | Vial |
| Amiodarone | 150 mg  200 mg | Vial  Tablet |
| Amitriptyline | 25 mg | Tablet |
| Amlodipine | 5 mg  10 mg | Tablet  Tablet |
| Amoxicillin | 250 mg/ 100 ml  500 mg  500 mg/ 100 ml | Suspension  Capsule  Suspension |
| Ampicillin | 500 mg  1 g  250 mg x 60 ml  500 mg | Vial  Vial  Suspension  Capsule |
| Anatoxina tetanica | 0.5 ml | Vial |
| Ascorbic acid | 100 mg/ml 30 ml  500 mg | Oral drops  Chewable tablet |
| Atorvastatin | 20 mg, 40 mg | Tablet |
| Atropine (sulphate) | 1 mg | Vial |
| Azithromycin | 200 mg x 15 ml  500 mg | Suspension  Tablet |
| Beclomethasone | 50 mcg  250 mcg | Nasal inhalator  Oral inhalator |
| Benzyl benzoate | 30% | Lotion |
| Betamethasone | 0,05%  3 mg  4 mg  8 mg | Cream  Vial  Vial  Vial |
| Betamethasone + Clotrimazole + Neomycin | 0.04% + 1% + 0.5 g | Cream |
| Beta-methyldigoxin | 0.1 mg  0.6 mg  2 ml | Tablet  Oral drops  Vial |
| Biperiden | 2 mg | Tablet |
| Brimonidine (tartrate) | 5 ml | Eye drops |
| Butylscopalamine bromide (Hioszinbutyl bromide) | 10 mg | Tablet |
| Calcium + Vitamin D | 600 + 200 IU | Tablet |
| Captopril | 25 mg  50 mg | Tablet  Tablet |
| Carbamazepine | 2%  200 mg | Oral suspension  Tablet |
| Carvedilol | 6.25 mg  12.5 mg  25 mg | Tablet  Tablet  Tablet |
| Cefalexin | 500 mg  250 mg/ 5ml | Capsule  Suspension |
| Cefradine | 500 mg  1 g | Tablet  Vial |
| Ceftriaxone | 1 g | Vial |
| Chloroquine | 250 mg | Tablet |
| Chlorphenamine | 2 mg/ 5 ml 120 ml  4 mg | Syrup  Tablet |
| Ciprofloxacin | 500 mg | Tablet |
| Clarithromycin | 250 mg  500 mg | Suspension  Tablet |
| Clonidine chlorhydrate | 150 mg | Tablet |
| Clopidogrel | 75 mg | Tablet |
| Clotrimazole | 1%  1%  100 mg  30 ml | Topical cream  Vaginal cream  Vaginal tablet  Lotion |
| Colchicine | 0.5 mg | Tablet |
| Colistin + Neomycin |  | Ear drops |
| Conjugated oestrogens | 0.625 mg | Tablet |
| Cotrimocazole (Trimethoprim + Sulfmethoxazole) | 160 mg + 800 mg  40 mg + 200 mg/ 5 ml 120 ml | Tablet  Suspension |
| Cromoglicate | 4%  4% | Nose drops  Eye drops |
| Crotamiton | 10% x 60 ml | Lotion |
| Dexamethasone | 4 mg  8 mg | Vial  Vial |
| Diclofenac | 50 mg  75 mg | Tablet  Vial |
| Dicloxacillin | 250 mg x 80 ml  500 mg | Suspension  Capsule |
| Dihydrocodeine | 2.42 120 ml | Syrup |
| Dimenhydrinate | 50 mg  50 mg | Tablet  Capsule |
| Diphenhydramine | 12.5 mg/ 5 ml 120 ml  100 mg | Syrup  Tablet |
| Doxycycline | 100 mg | Tablet |
| Enalapril | 5 mg  20 mg | Tablet  Tablet |
| Ergotamine + Caffeine | 1 mg + 100 mg | Tablet |
| Erythromycin | 500 mg  250 mg | Tablet  Suspension |
| Esomeprazole | 20 mg  40 mg | Tablet |
| Fluconazole | 200 mg  50 mg/ 5 ml 20 ml | Tablet  Suspension |
| Fluoxetine | 20 mg | Tablet |
| Folic acid | 1 mg | Tablet |
| Furosemide | 40 mg | Tablet |
| Gemfibrozil | 600 mg | Tablet |
| Gentamicin | 0,3%  80 mg | Eye drops  Vial |
| Glibenclamide | 5 mg | Tablet |
| Haloperidol | 5 mg | Tablet |
| Hartmann solution | 500 ml | Solution for injection |
| Hydrochlorothiazide | 25 mg | Tablet |
| Hydrocortisone | 0.5%  1%  100 mg | Lotion  Topical cream  Vial |
| Hydrocortisone + Benzocaine |  | Rectal cream |
| Hydroxyzine | 100 mg | Vial |
| Ibuprofen | 400 mg | Tablet |
| Ipratropium bromide |  | Inhalator |
| Iron fumarate + folic acid +ascorbic acid (vitamin c) |  | Tablet |
| Iron sulphate | 125 mg/ ml 20 ml  300 mg  600 mg / 15 ml 120 ml | Oral drops  Tablet  Syrup |
| Isosorbide | 5 mg | Sublingual tablet |
| Isosorbide dinitrate | 10 mg | Tablet |
| Ketoconazole | 100 mg 60 ml  200 mg | Suspension  Tablet |
| Ketotifen | 1 mg  1 mg/ 5 ml x 100 ml | Tablet  Syrup |
| Levodopa + Carbidopa | 250 mg + 25 mg | Tablet |
| Levomepromazine | 25 mg  100 mg  4% | Tablet  Tablet  Oral drops |
| Levonorgestrel | 0.03 mg | Tablet |
| Levonorgestrel + Ethinylestradiol | 100 mcg + 20 mcg | Tablet |
| Levothyroxine | 25 mcg  50 mcg  100 mcg  125 mcg  150 mcg | Tablet  Tablet  Tablet  Tablet  Tablet |
| Lidocaine chlorohydrate | 2% | Gel |
| Loratadine | 5 mg/ 5 ml x 100 ml  10 mg | Syrup  Tablet |
| Losartan | 50 mg  100 mg | Tablet  Tablet |
| Losartan + Hydrochlorothiazide | 100 mg + 25 mg | Tablet |
| Mebendazole | 100 mg  100 mg | Suspension  Tablet |
| Medroxyprogesterone + Oestradiol | 25 mg + 5 mg | Vial |
| Medroxyprogesterone acetate | 50 mg/ ml (5%) | Vial |
| Metamizole | 1g/ 2 ml  2.5 g | Vial  Vial |
| Metformin | 850 mg | Tablet |
| Methocarbamol | 750 mg | Tablet |
| Methotrexate | 2.5 mg | Tablet |
| Methylprednisolone | 500 mg | Vial |
| Metoclopramide | 10 mg  10 mg | Tablet  Vial |
| Metoprolol succinate | 25 mg  50 mg  100 mg | Tablet  Tablet  Tablet |
| Metoprolol tartrate | 50 mg  100 mg | Tablet  Tablet |
| Metronidazole | 250 mg/ 5 ml  500 mg  500 mg | Suspension  Ovule  Tablet |
| Nalidixic acid | 250 mg/ 120 ml | Suspension |
| Naproxen | 2.5% 80 ml | Suspension |
| Naproxen + Caffeine | 220 mg + 50 mg | Tablet |
| Neomycin + Polymyxin + Dexamethasone |  | Eye drops |
| Nifedipine | 10 mg  30 mg | Capsule  Tablet |
| Nimodipine | 30 mg | Tablet |
| Nitrofurantoin | 100 mg | Capsules |
| Nitrofurazone | 500 mg | Topical cream |
| Norfloxacin | 400 mg | Tablet |
| Nystatin | 100,000 IU  100,000 IU  500,000 IU | Suspension  Vaginal tablet  Tablet |
| Nystatin + Zinc oxide |  | Topical cream |
| Olanzapine | 5 mg  10 mg | Tablet  Tablet |
| Omeprazole | 20 mg | Capsule |
| Oxacillin | 1 g | Vial |
| Oxymetazoline | 0.25%, 0.05% | Nasal drops |
| Paracetamol | 100 mg/ml x 30ml  150 mg/ml x 90 ml  500 mg | Oral drops  Syrup  Tablet |
| Penicillin | 1,000,000 IU  1,200,000 IU  2,400,000 IU | Vial  Vial  Vial |
| Phenytoin | 100 mg  250 mg/ 5ml | Tablet  Vial |
| Physiological serum | 500 ml | Bag  Nasal drops |
| Pipotiazine | 25 mg/ 1ml | Vial |
| Potassium chloride | 10 ml | Vial |
| Prednisolone | 5 mg | Tablet |
| Propranolol | 40 mg  80 mg | Tablet  Tablet |
| Pyrantel pamoate | 250 mg  250 mg/ 5ml x 15 ml | Tablet  Suspension |
| Ranitidine | 300 mg  50 mg | Tablet  Vial |
| Risperidone | 1 mg | Tablet |
| Salbutamol | 100 mcg  2 mg/ 5ml 120 ml | Inhalator  Salt to nebulize  Syrup |
| Salts for oral rehydration |  | Bag |
| Silver sulphadiazine | 1% | Cream |
| Sodium chloride | 10 ml | Vial |
| Spironolactone | 25 mg  100 mg | Tablet  Tablet |
| Sucralfate | 1g | Tablet |
| Theophylline | 125 mg  80 mg/ 15 ml | Tablet  Elixir |
| Timolol maleate | 0.5% | Eye drops |
| Tinidazole | 500 mg | Tablet |
| Tramadol | 100 mg/ 10 ml  100 mg/ ml | Oral drops  Vial |
| Tranexamic acid | 500 mg | Tablet |
| Trazodone | 50 mg | Tablet |
| Trimebutine | 200 mg | Tablet |
| Valproate | 250 mg  250 mg/ 5ml 120 ml | Soft capsule  Syrup |
| Valsartan | 80 mg  160 mg | Tablet  Tablet |
| Valsartan + Hydrochlorothiazide | 160 mg + 12.5 mg  80 mg + 12.5 mg | Tablet  Tablet |
| Vecuronium bromide | 10 mg | Vial |
| Verapamil | 80 mg  120 mg  240 mg | Tablet  Tablet  Tablet |
| Vitamin A (Retinol) | 50,000 IU | Soft capsule |
| Vitamin B1 (Thiamine) | 10 ml  300 mg | Vial  Tablet |
| Vitamin B12 | 2 ml | Vial |
| Vitamin K | 10 mg | Vial |
| Warfarin sodica | 5 mg | Tablet |
| Zinc sulphate | 2 mg/ 5ml 120 ml | Syrup |
